# Structured nucleosome fingerprints enable high-resolution mapping of chromatin architecture within regulatory regions

**DOI:** 10.1101/gr.192294.115

**Published:** 2015-11

**Authors:** Alicia N. Schep, Jason D. Buenrostro, Sarah K. Denny, Katja Schwartz, Gavin Sherlock, William J. Greenleaf

**Affiliations:** 1 Department of Genetics, Stanford University School of Medicine, Stanford, California 94305, USA;; 2 Biophysics Program, Stanford University School of Medicine, Stanford, California 94305, USA;; 3 Department of Applied Physics, Stanford University, Stanford, California 94305, USA

## Abstract

Transcription factors canonically bind nucleosome-free DNA, making the positioning of nucleosomes within regulatory regions crucial to the regulation of gene expression. Using the assay of transposase accessible chromatin (ATAC-seq), we observe a highly structured pattern of DNA fragment lengths and positions around nucleosomes in *Saccharomyces cerevisiae*, and use this distinctive two-dimensional nucleosomal “fingerprint” as the basis for a new nucleosome-positioning algorithm called NucleoATAC. We show that NucleoATAC can identify the rotational and translational positions of nucleosomes with up to base-pair resolution and provide quantitative measures of nucleosome occupancy in *S. cerevisiae*, *Schizosaccharomyces pombe*, and human cells. We demonstrate the application of NucleoATAC to a number of outstanding problems in chromatin biology, including analysis of sequence features underlying nucleosome positioning, promoter chromatin architecture across species, identification of transient changes in nucleosome occupancy and positioning during a dynamic cellular response, and integrated analysis of nucleosome occupancy and transcription factor binding.

Chromatin accessibility modulates the ability of transcription factors (TFs) and transcriptional machinery to interact with DNA. Within regions of increased accessibility, nucleosomes and TFs compete for access to regulatory DNA (Lickwar et al. 2012). While sequence content has been shown to influence nucleosome positioning, the specific locations of nucleosomes in vivo are also dynamically modulated by chromatin remodelers, transcription factors, and transcriptional machinery (Kaplan et al. 2009; Zhang et al. 2009; Valouev et al. 2011). Therefore, methods for producing base-pair resolved nucleosome maps with quantitative occupancy information within regulatory DNA promise to provide insight into the interplay between chromatin organization and transcriptional regulators—a crucial step toward a comprehensive and predictive understanding of how regulatory elements control gene expression.

Methods for inferring nucleosome positions through MNase digestion of chromatin followed by high-throughput sequencing of protected fragments have provided a window into the relationship between TF binding and nucleosome positioning, revealing that certain TFs are consistently flanked by well-positioned nucleosomes, while others show considerable heterogeneity in the positioning of proximal nucleosomes (Valouev et al. 2011; Gaffney et al. 2012; Kundaje et al. 2012). However, MNase-based methods are limited in their ability to infer high-resolution nucleosome positions and provide quantitative measures of nucleosome occupancy by the enzyme's processive nature of DNA digestion and intrinsic digestion sequence bias (Chung et al. 2010; Fan et al. 2010). Alternatively, chemical mapping approaches in both *Saccharomyces cerevisiae* and *Schizosaccharomyces pombe* have provided base-pair resolved maps of nucleosome positions (Brogaard et al. 2012; Moyle-Heyrman et al. 2013). However, this high-resolution chemical cleavage technique cannot be easily adapted to other biological systems, as it requires a genetically modified histone H4. Furthermore, as with MNase-based assays, chemical mapping has limited ability to measure absolute nucleosome occupancy, as nucleosome depletion is indirectly inferred through lack of signal.

We recently described the assay for transposase-accessible chromatin using sequencing (ATAC-seq), a method for rapid, sensitive, genome-wide profiling of chromatin accessibility (Buenrostro et al. 2013). Here, we adapt ATAC-seq to *S. cerevisiae* and discover a highly structured, reproducible ATAC-seq fragmentation pattern around nucleosomes. We use this “nucleosome fingerprint” as the basis of NucleoATAC, a computational method for quantitative, high-resolution inference of nucleosome positioning and occupancy within regulatory regions. We highlight several applications of NucleoATAC by examining differences in chromatin architecture in regulatory regions between *S. cerevisiae*, *S. pombe*, and human, elucidating changes in nucleosome positioning and occupancy during a dynamic transcriptional response in yeast, and determining nucleosome occupancy and positioning relative to transcription factors in a human lymphoblastoid cell line.

## Results

### V-plots demonstrate structured ATAC-seq signal around nucleosomes

**Figure 1.**
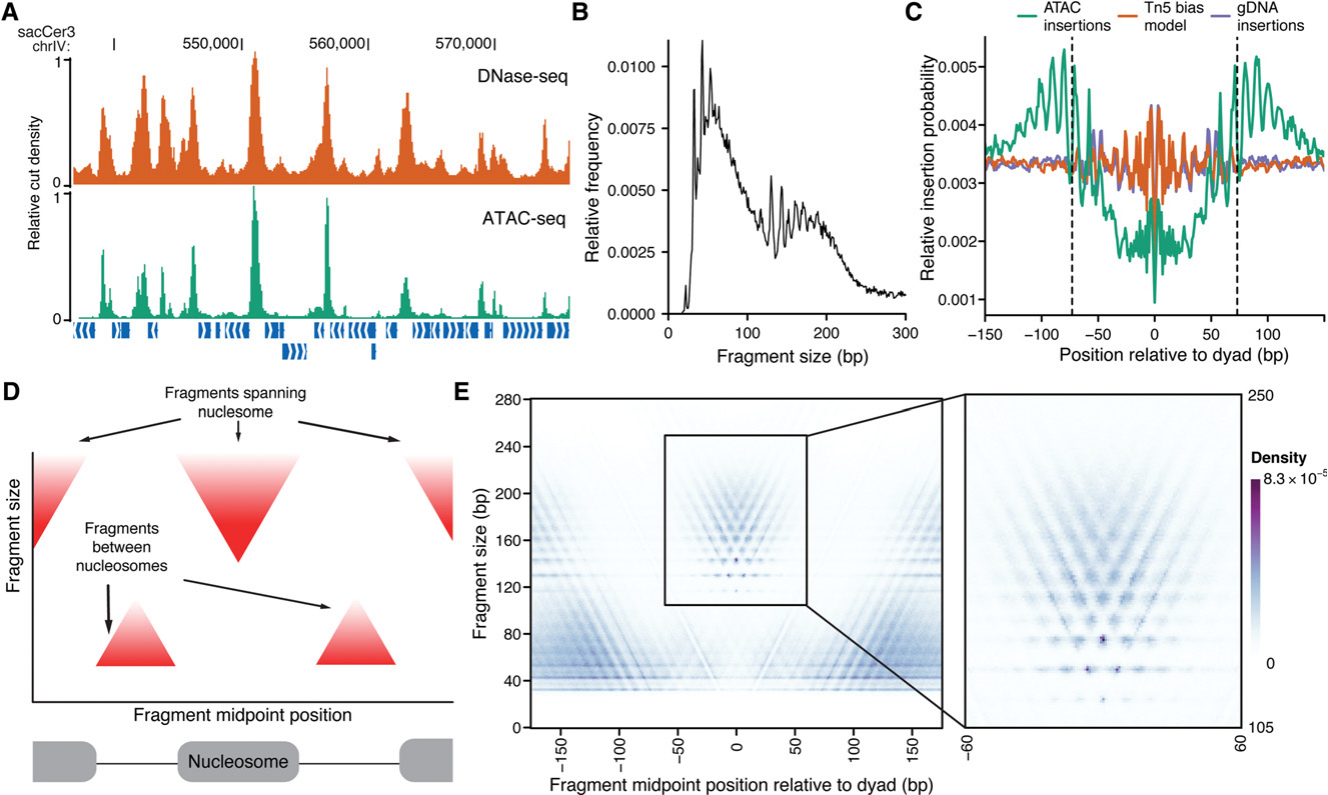
ATAC-seq signal is highly structured around nucleosomes. (*A*) ATAC-seq (green) insertion track for *S. cerevisiae* shows enrichment of insertions at accessible chromatin regions, similar to DNase-seq cut density (orange). Both tracks were smoothed by 150 bp and scaled so that the maximum density in the region is 1. (*B*) Fragment-size distribution for *S. cerevisiae* ATAC-seq samples. (*C*) Insertion probabilities for ATAC-seq (teal), genomic DNA (purple), and predicted by sequence bias (orange) (see Methods) around nucleosomes defined by chemical mapping. (*D*) Schematic illustration of expected V-plot pattern around a well-positioned nucleosome. (*E*) V-plot (fragment size versus fragment center position) of ATAC-seq fragments around well-positioned nucleosomes called by chemical mapping, with *inset* showing region with nucleosome-spanning fragments.

We previously observed that short ATAC-seq fragments are concentrated at nucleosome-free regions (NFR), whereas long fragments are enriched at nucleosome-associated DNA (Buenrostro et al. 2013). To further examine this association, we developed an *S. cerevisiae* ATAC-seq protocol to determine ATAC-seq fragmentation patterns at positions of base-pair resolved nucleosomes in *S. cerevisiae* generated using chemical mapping techniques (Brogaard et al. 2012). Using ATAC-seq for *S. cerevisiae*, we generated 61 million paired-end ATAC-seq reads with high mapping quality across 11 replicates, which were highly reproducible (Supplemental Fig. 1; Methods). ATAC-seq read depth for *S. cerevisiae* is highly correlated with DNase-seq (Fig. 1A; Supplemental Fig. 2A; Hesselberth et al. 2009) but shows greater enrichment in promoters (Supplemental Fig. 2B), demonstrating that ATAC-seq provides a sensitive measure of chromatin accessibility genome-wide. As with mammalian ATAC-seq, fragment sizes for *S. cerevisiae* reflect nucleosome organization, with a peak in the fragment-size distribution at 140–200 bp arising from DNA protected by a nucleosome (Fig. 1B), although peaks for multiple nucleosomes (e.g., di- or trinucleosomes) are much weaker or not observable.

By aggregating ATAC-seq transposition centers around well-positioned, base-pair resolved nucleosome positions determined by chemical mapping (Brogaard et al. 2012), we observe clear protection from transposase insertion within nucleosomal DNA (Fig. 1C). Additionally, we observe striking periodicity in the insertions at the boundary of the nucleosome. We postulate that this periodicity arises from steric hindrance of the Tn5 transposase at the nucleosome boundary, which allows for only one face of the DNA double-helix to be accessible to transposition. To further characterize the ATAC-seq signal around these nucleosome dyad positions, we mapped fragment midpoints and sizes using a “V-plot” (Fig. 1D; Henikoff et al. 2011). This visualization maps the density of fragment sizes versus fragment center locations relative to a genomic feature of interest (in this case, nucleosome dyads). These aggregate protection profiles show a V-shaped structure, where the apex of the “V” represents the smallest possible fragment that spans the DNA protected by a nucleosome. The V-plot centered on chemically mapped dyads shows a clear depletion of short fragments in the portion of DNA wrapped around the nucleosome (Fig. 1E). At fragment sizes spanning a nucleosome (Fig. 1E, inset), we observe a highly structured V-pattern with both horizontal and vertical periodicity. This periodicity likely reflects both the steric hindrance of the transposase (vertical and horizontal periodicity) and previously described 10-bp rotational positioning of nucleosomes in yeast (horizontal periodicity). The apex of the V shape is at 117 bp, while the most abundant position in the V-plot represents fragments of 143 bp centered at the dyad. These smaller-than-expected fragment sizes may arise from stochastic “breathing” of DNA associated with nucleosomes, allowing for transposase insertions within the 147 bp that are canonically considered to be nucleosome-associated (Anderson et al. 2002) or from nucleosomes packed closer than 147 bp apart (Chereji and Morozov 2014).

### Determining nucleosome positions from structured V-plot

**Figure 2.**
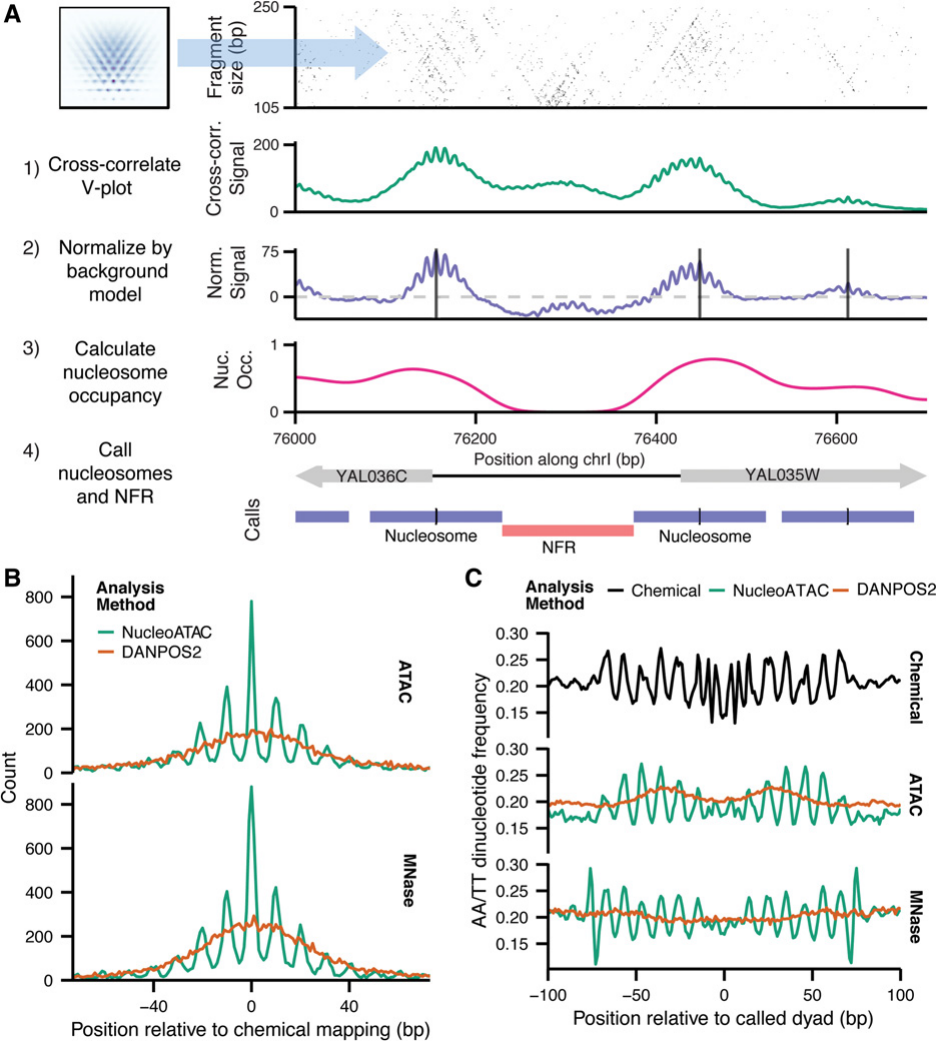
NucleoATAC enables high-resolution nucleosome positioning. (*A*) Schematic of NucleoATAC workflow. First, the V-plot nucleosome signature is cross-correlated against a 2D fragment size versus fragment midpoint representation of ATAC-seq data at a locus. The signal is then normalized by a background model (based on sequence bias and read depth) to obtain a normalized signal. Nucleosome occupancy is calculated using the local fraction of nucleosomal fragments. The normalized cross-correlation signal and nucleosome occupancy tracks are used to assign nucleosome and nucleosome-free (NFR) positions. (*B*) Distance of dyad calls from different assays (ATAC on *top* panel; MNase on *bottom*) using either NucleoATAC (green) or DANPOS2 (orange). (*C*) AA/TT dinucleotide pattern around nucleosome dyad calls determined by chemical mapping (*top* panel), or from ATAC-seq (*middle* panel), or MNase-seq (*bottom* panel) using either NucleoATAC (green) or DANPOS2 (orange).

We reasoned that standard methods for inferring nucleosome centers, which assume that fragment midpoints are normally distributed around the nucleosome core (Chen et al. 2013; Polishko et al. 2014), could be improved by leveraging this highly structured two-dimensional V-plot pattern. To this end, we developed NucleoATAC (Fig. 2), an algorithm that cross-correlates the characteristic, average nucleosome V-plot against a V-plot representation of fragments across regions of the genome (see Methods). This cross-correlation signal measures how well ATAC-seq data at any particular base fits the expected pattern at a nucleosome dyad. To account for the possibility of spurious signal from Tn5 insertion sequence bias (Adey et al. 2010; Buenrostro et al. 2013) and signal variation based on differential chromatin openness, we normalize this nucleosome signal by subtracting a calculated background signal expected from transposition sequence bias, the global fragment-size distribution of the sample, and the number of fragments in the region. Peaks from the background-subtracted signal track are used to identify dyad positions, which are then scored for several characteristics that can be used for downstream filtering (see Methods; Supplemental Fig. 3). This background-subtracted cross-correlation signal provides high-resolution positional information regarding the location of nucleosomes, but it is correlated with fragment coverage and therefore cannot be used for accurately determining nucleosome occupancy. We therefore developed a method for estimating nucleosome occupancy in which the global fragment-size distribution is modeled as a mixture of two distributions, nucleosomal and nucleosome-free (Supplemental Figs. 4, 5; Supplemental Note 1), and the maximum likelihood fraction of nucleosomal reads at a locus is taken as the occupancy score.

### NucleoATAC enables high-resolution nucleosome calling in *S. cerevisiae*

NucleoATAC identified the positions of 13,344 nucleosomes across broad open chromatin regions in the yeast genome (with *Z*-score ≥ 3, log-likelihood ratio > 0) (see Methods), compared to 17,015 positions determined across these same regions using chemical mapping. Fewer calls are made by NucleoATAC relative to chemical mapping because ATAC-seq coverage varies greatly across the genome based on accessibility; however, ATAC-seq coverage by short fragments can be used to distinguish between genuine nucleosome depletion and absence of nucleosome calls due to low accessibility (Supplemental Note 2; Supplemental Fig. 6). We found that no characteristic nucleosome fingerprint is observed when aggregating ATAC-seq insertions generated from genomic DNA or predicted by transposase bias signal (Supplemental Fig. 7) around NucleoATAC-called nucleosomes, suggesting residual intrinsic Tn5 insertion bias has little effect on aggregate NucleoATAC nucleosome calls.

Nucleosome positioning calls determined by NucleoATAC are highly concordant (Table 1; Fig. 2B) with chemically mapped nucleosomes (Brogaard et al. 2012), and divergent calls are generally offset by multiples of 10 bp. We quantified a number of positional concordance metrics for nucleosome calls using our method (distance AUC, sensitivity, and specificity) (see Methods). We also tested a method of nucleosome calling by splitting reads based on fragment size and then using DANPOS2 (Chen et al. 2013), similar to the method previously used for calling nucleosomes with ATAC-seq (Buenrostro et al. 2013). NucleoATAC outperforms the DANPOS2 method on all metrics (Table 1; Supplemental Table 1). We also quantified the “rotational specificity” of each set of calls, defined as the fraction of nucleosome calls within 1 bp of a call in the redundant nucleosome map derived from chemical mapping. The redundant map includes nucleosome positions that overlap and shows that overlapping nucleosome positions are often offset by multiples of 10 bp; concordance with this map suggests that in vivo nucleosome positions are being precisely captured. For calls made by NucleoATAC, 34% of positions determined a match within a 1 bp position from the redundant map. This rotational positioning enables us to observe the underlying sequence periodicity that may dictate the rotational positioning of nucleosomes; AA/TT dinucleotide content exhibits periodicity within DNA contacting NucleoATAC nucleosomes similar to that within nucleosomes called by chemical mapping (Fig. 2C).

**Table 1.**
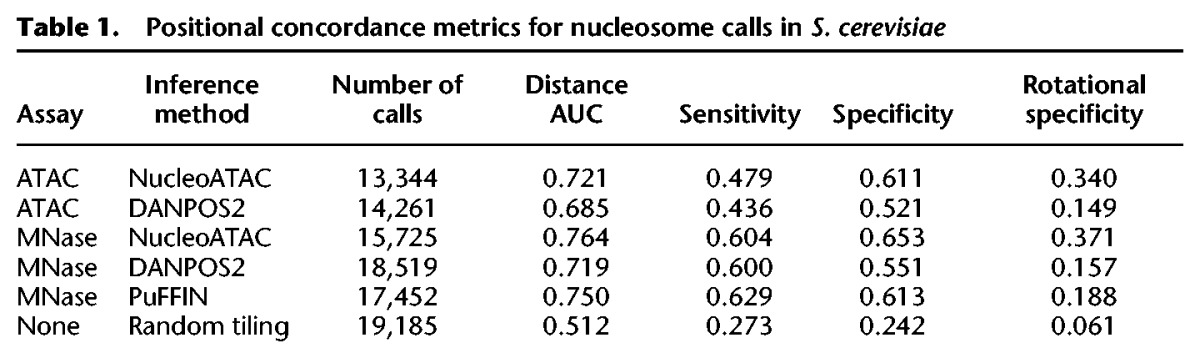
Positional concordance metrics for nucleosome calls in *S. cerevisiae*

The confidence metrics used for filtering NucleoATAC calls enable the algorithm to be robust to sequencing depth; when down-sampling or using individual replicates from our data, fewer nucleosomes are called, but called nucleosomes have similar concordance with the chemical mapping calls (Supplemental Figs. 8,9). As positional concordance between NucleoATAC and chemical mapping calls increases as a function of both NucleoATAC and chemical map confidence metrics (Supplemental Fig. 10), discrepancies between the two methods are likely partially due to either lower quality chemical mapping calls or inconsistently positioned nucleosomes.

We also sought to determine whether the cross-correlation analysis in NucleoATAC might be applicable to MNase data sets as well, using V-plots derived from paired-end MNase data sets (Supplemental Fig. 10). NucleoATAC applied to MNase data sets is also able to capture the rotational positioning of nucleosomes (Fig. 2B,C) and outperforms other methods for calling nucleosome positions using MNase (Table 1; Supplemental Table 2; Supplemental Fig. 10), although we observe that the V-plot pattern observed appears to be sensitive to the MNase protocol used (Supplemental Fig. 11). In contrast, the V-plot pattern appears consistent between ATAC-seq samples, even when using a different spheroplasting protocol and transposase incubation time (Supplemental Fig. 12).

### NucleoATAC can be applied across species

**Figure 3.**
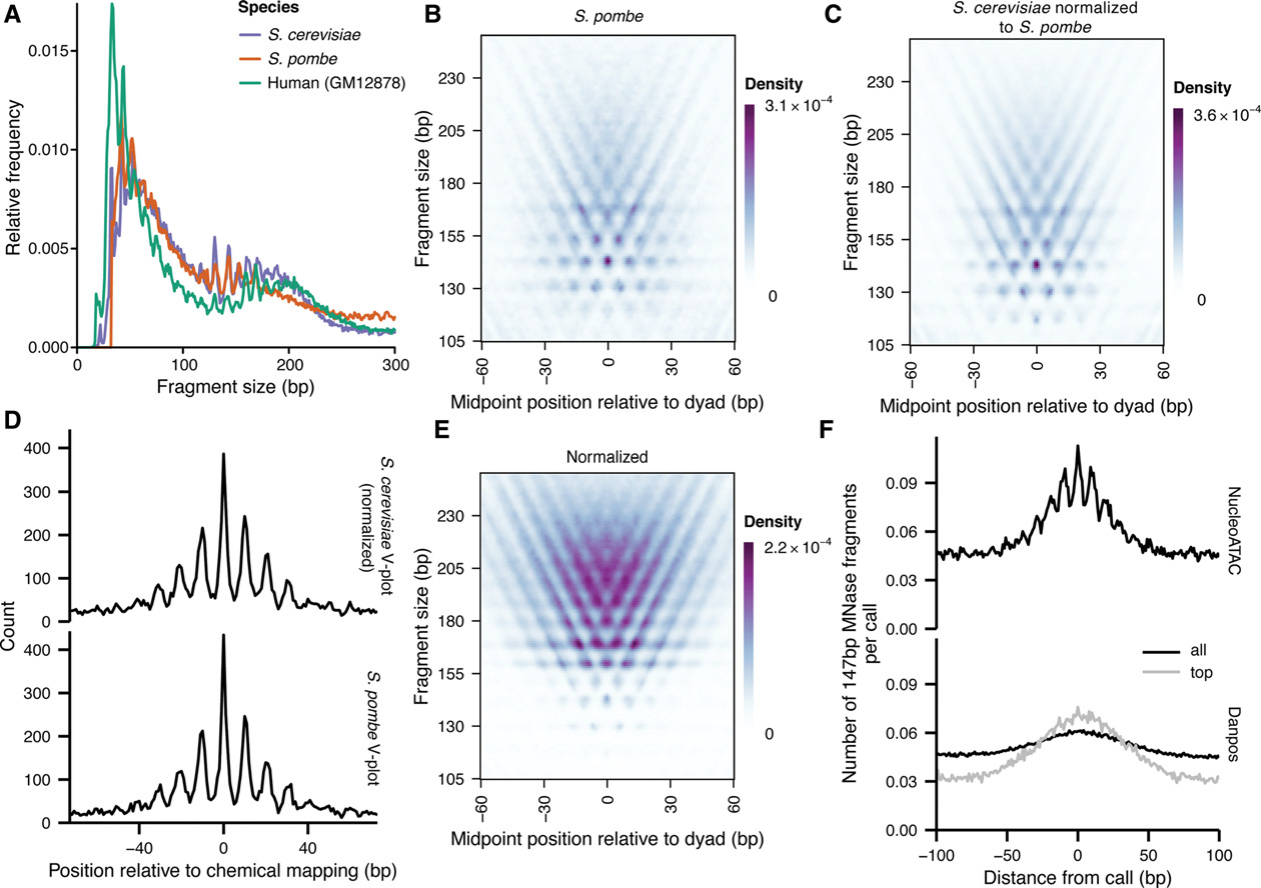
V-plot derived from *S. cerevisiae* can be used as a template to apply NucleoATAC to other species. (*A*) Fragment-size distributions for *S. cerevisiae* (purple), *S. pombe* (orange), and human GM12878 cell line (teal). (*B*) *S. pombe* V-plot based on chemical map calls for *S. pombe*. (*C*) *S. cerevisiae* V-plot normalized to match *S. pombe* fragment-size distribution. (*D*) Comparison of NucleoATAC concordance with chemical mapping for *S. pombe* when using V-plots in *B* or *C*. (*E*) *S. cerevisiae* V-plot normalized to match human GM12878 fragment sizes. (*F*) 147-bp MNase fragment density around calls for GM12878 made by either NucleoATAC (black curve in *upper* panel) or DANPOS (black and gray curves in *lower* panel; gray curve is restricted to top calls to match the number of calls made by NucleoATAC).

**Figure 4.**
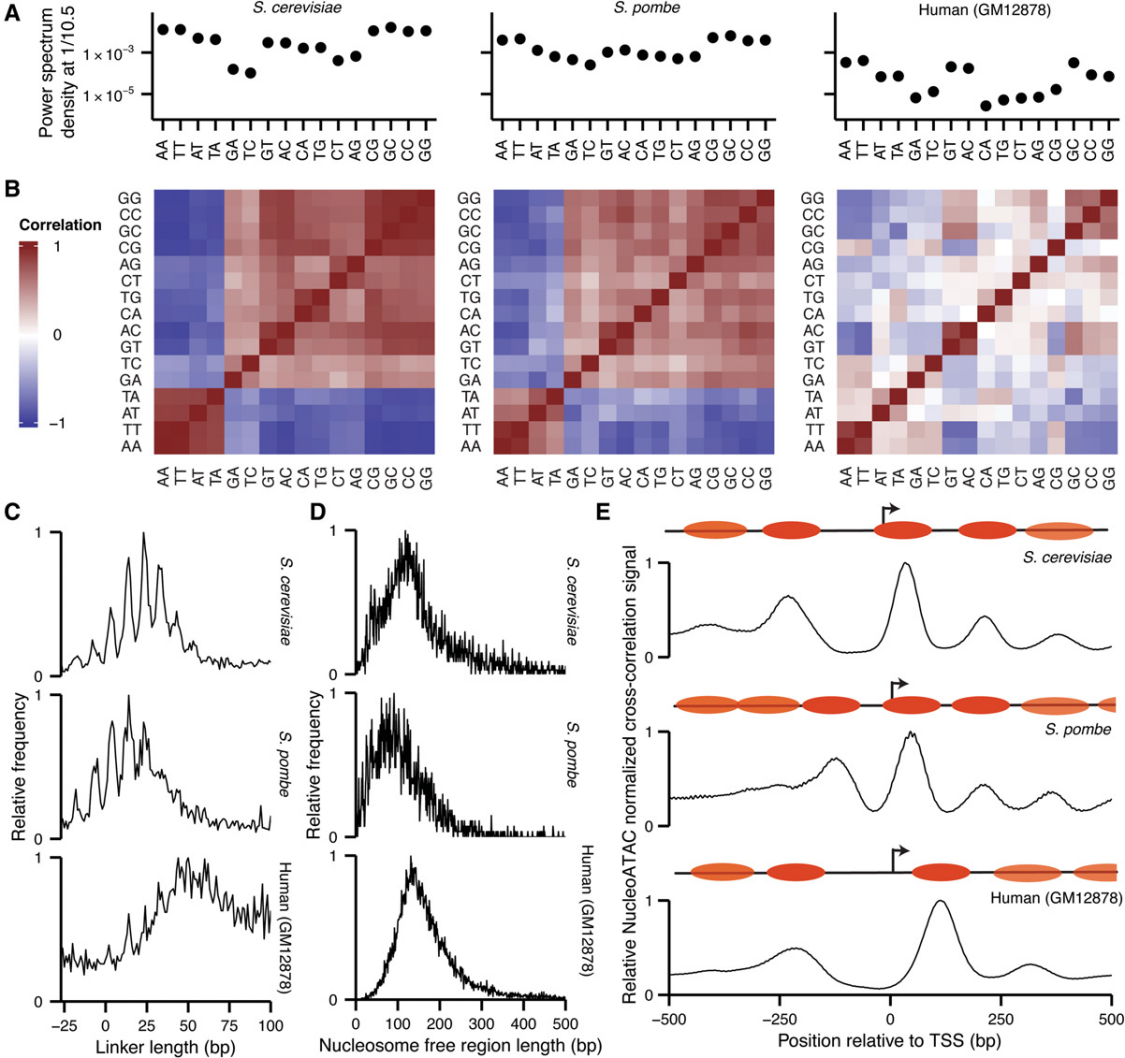
NucleoATAC reveals differences in nucleosome architecture between species. (*A*) Power spectrum density at 1/10.5 bp for each dinucleotide from 19 to 60 bp from NucleoATAC-called dyads for *S. cerevisiae*, *S. pombe*, and human (*left* to *right*). (*B*) Pair-wise correlation between dinucleotide frequencies for each species. (*C*) Distances between adjacent nucleosomes in three species. (*D*) Nucleosome-free region lengths for three species. (*E*) Positive NucleoATAC cross-correlation signal aggregated at TSS in three species. Cartoons show canonical nucleosome positioning at TSS for each species, with more transparent nucleosome ovals representing nucleosomes that are less consistently positioned among different TSSs.

Because histones are among the most evolutionarily conserved proteins, we hypothesized that the same structured V-plot “nucleosome fingerprint” pattern is present for different species. To test this possibility, we first developed ATAC-seq for *Schizosaccharomyces pombe*, a species highly diverged from *S. cerevisiae* (Rhind et al. 2011) for which high-resolution chemical mapping data are also available (Moyle-Heyrman et al. 2013). Subtle aspects of the ATAC-seq fragment-size distribution for *S. pombe* differed from *S. cerevisiae*, as might be expected based on previously characterized differences in average linker lengths (Moyle-Heyrman et al. 2013) and variation in fragment size observed between different ATAC-seq samples of the same species (Supplemental Fig. 13). However, local maxima in the distributions aligned well (Fig. 3A), suggesting similar nucleosomal constraints on Tn5 insertion between the species. We reasoned that adjusting the *S. cerevisiae* V-plot such that the summed intensity of each row would match the nucleosomal fragment-size distribution of the *S. pombe* sample (see Methods) would approximate the *S. pombe* nucleosome V-plot. Indeed, the resulting V-plot is similar to a V-plot made from ATAC-seq reads using *S. pombe* chemically mapped dyads (*R* = 0.96 after normalization; *R* = 0.81 without normalization) (Fig. 3B,C). We applied NucleoATAC using both the adjusted *S. cerevisiae* V-plot and the “true” V-plot from *S. pombe* chemical map calls and found the resulting calls to be very similar (distance AUC = 0.97) (Supplemental Fig. 14) and similarly concordant with the chemical mapping calls **(**Table 2; Fig. 4D). We also tested whether the V-plot from *S. pombe* could be used for *S. cerevisiae*; positional concordance with chemical mapping calls for *S. cerevisiae* is similar when using either the *S. cerevisiae* V-plot or the fragment size normalized *S. pombe* V-plot (Table 2). These results suggest that nucleosomal constraints on the Tn5 transposase are conserved across species and that NucleoATAC (using the V-plot from *S. cerevisiae*) may be applied to diverse organisms of interest.

**Table 2.**
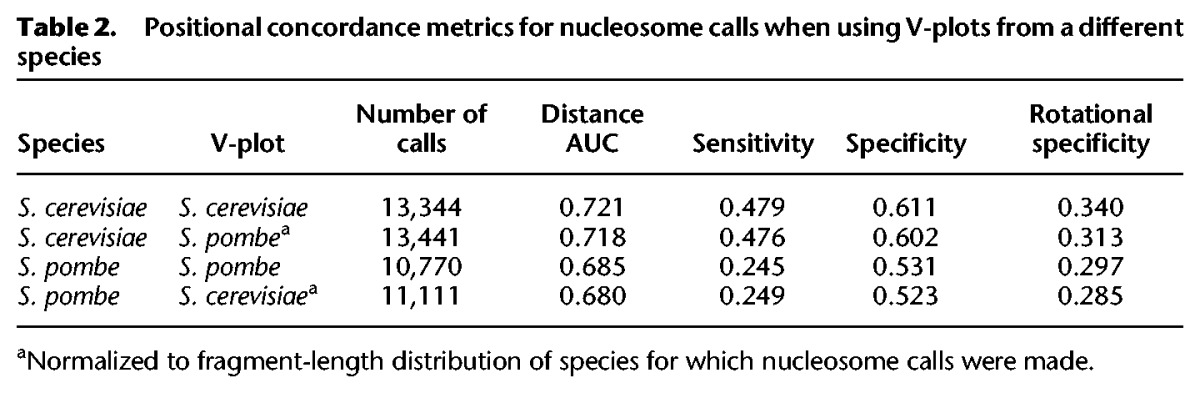
Positional concordance metrics for nucleosome calls when using V-plots from a different species

As with *S. pombe*, the local maxima in the fragment-size distribution for ATAC-seq data from the human lymphoblastoid GM12878 cell line (Buenrostro et al. 2013) are similar to that observed for *S. cerevisiae* (Fig. 3A). Thus, to apply NucleoATAC to these data, we similarly normalized the V-plot signal density from *S. cerevisiae* to match the nucleosomal fragment-size distribution of the human data (Fig. 3E). To assess our ability to capture translational and rotational positioning of human nucleosomes, we compared our calls with reported MNase fragment center positions (Gaffney et al. 2012). We focused on 147-bp fragments, as the previous work has shown that these fragments are enriched for fragments that precisely span a single nucleosome and that these fragments therefore provide a high-resolution measure of nucleosome positions (Gaffney et al. 2012). The positions of these 147-bp MNase fragment centers are enriched at NucleoATAC calls and display clear 10-bp periodicity (Fig. 3F), validating our ability to capture rotational information in human cells.

### Comparison of nucleosome positioning across species

High-resolution NucleoATAC nucleosome calls and signal tracks allowed for a comprehensive and quantitative analysis of sequence preferences of well-positioned nucleosomes in regulatory regions across these species. Dinucleotide frequencies between 19 and 60 bp from the nucleosome dyad often displayed a 10.5-bp periodicity, tracking the helical pitch of DNA around the nucleosome (Supplemental Fig. 15). To quantify the strength of the 10.5-bp oscillations for each dinucleotide, we computed the power spectrum density at frequency 1/10.5 bp (Fig. 4A), providing a measure of the intensity of this 10.5-bp periodicity. We also computed a pair-wise correlation of dinucleotide frequencies between every dinucleotide for each species (Fig. 4B). For the two yeast species, all dinucleotides show 10.5-bp periodicity, with the AA, TA, AT, and TT dinucleotides being out of phase with all the other dinucleotides. For human nucleosomes, we observe overall much smaller magnitude periodicity, with the strongest relative periodicity in AA, TT, GT, AC, GC, CC, and GG, and only weak or negligible power for other dinucleotides. The weaker periodicity in CG relative to GC, GG, and CC might reflect the effect of CpG methylation, in line with other work suggesting that methylated CpG frequencies oscillate out of phase with unmethylated CG (Collings et al. 2013).

Our high-resolution calls allow for a detailed comparison of regulatory architecture across these three disparate species. Linker length can be computed by determining the distance between adjacent calls; linker length varies between the species, with *S. pombe* having the shortest linker length and human the longest, consistent with previous observations (Tsankov et al. 2011; Moyle-Heyrman et al. 2013). For both yeast species, we observe “negative” linker lengths representing two nucleosomes with dyads being closer than 147 bp (Fig. 4C), supporting findings from paired-end chemical mapping and the hypothesis that chromatin can exist in a state with partially unwrapped nucleosomes (Chereji and Morozov 2014). We also determine nucleosome-free region lengths by identifying regions of low nucleosome occupancy between nucleosome calls (Fig. 4D). NFR lengths show similar trends as linker lengths, and for all species, nucleosome-free regions are generally smaller than the length occupied by a single nucleosome.

We aggregated NucleoATAC nucleosome signals around transcription start sites (TSSs) (Fig. 4E) to explore species-specific promoter architecture at high resolution. Nucleosome signals proximal to TSSs for both *S. cerevisiae* and humans show a clear depletion at the TSS, with the gap between the +1 and −1 nucleosome slightly larger for the human data. In contrast, this distance is similar to that between adjacent nucleosomes in *S. pombe*; a clear nucleosome-free region is not evident, consistent with results from chemical mapping (Moyle-Heyrman et al. 2013) but not with previous results from MNase that showed a pronounced NFR similar to that observed for *S. cerevisiae* (Lantermann et al. 2010). Others have attributed this discrepancy to the sequence bias of MNase (Moyle-Heyrman et al. 2013), as the AT-rich promoters of *S. pombe* are particularly sensitive to MNase digestion. We also mapped nucleosome occupancy for individual TSSs to determine whether there was heterogeneity in positioning of the +1/−1 nucleosomes that was being masked in the aggregate plot (Supplemental Fig. 16). We observe that the pattern observed in aggregate is present for the majority of individual TSS for each organism, although a small fraction of promoters in *S. pombe* do show a larger nucleosome-free region, and a fraction of promoters in *S. cerevisiae* and human lack a clear nucleosome-free region.

### Dynamic chromatin rearrangements during the osmotic stress response

**Figure 5.**
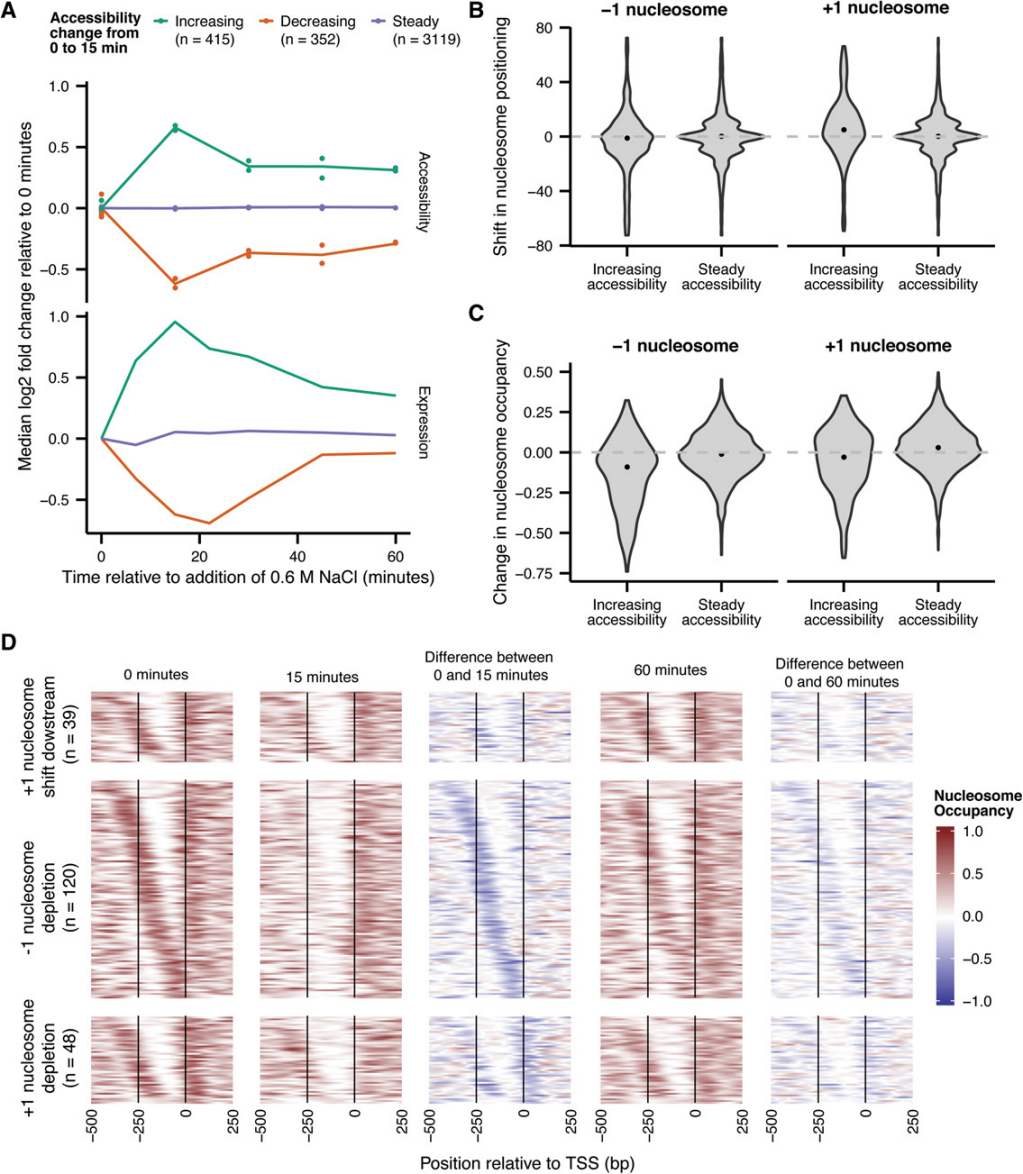
NucleoATAC reveals dynamics of nucleosome positioning and occupancy during osmotic stress response. (*A*) Promoter accessibility (*top*) and expression (*bottom*) changes over the osmotic stress time-course for genes showing an increase in accessibility from 0 to 15 min (green), a decrease in accessibility from 0 to 15 min (orange), or no significant change in accessibility between 0 and 15 min (purple). (*B*) Distribution of −1 and +1 nucleosome shifts for promoters with increasing accessibility and promoters with steady accessibility. (*C*) Distribution of −1 and +1 nucleosome occupancy changes for promoters with increasing accessibility and promoters with steady accessibility. (*D*) Individual occupancy traces for genes with significantly increased accessibility and characterized by either (1) downstream shifts in nucleosome positioning, (2) depletion of the −1 nucleosome, or (3) depletion of the +1 nucleosome during the first 15 min of the osmotic stress response. These categories do overlap.

To investigate the ability of NucleoATAC to infer nucleosome positioning and occupancy changes during a dynamic process, we performed ATAC-seq on yeast exposed to osmotic stress (0.6 M increase in the NaCl concentration over 60 min). Because osmotic stress induces transient gene expression changes that peak after 15 min (Ni et al. 2009), we identified promoters with significantly changed accessibility after 15 min (767 promoters with FDR < 0.01) (Supplemental Fig. 17). In aggregate, the accessibility at these promoters returned closer to steady-state levels during the time-course (Fig. 5A), mirroring gene expression changes for these promoters (Ni et al. 2009). Four hundred and fifteen promoters showed significant increases in accessibility, and these promoters are strongly enriched for GO terms associated with stress response, including oxidative stress and osmotic stress response (*P* < 10^−4^) (Supplemental Table 3). Furthermore, promoters that had both increased expression and increased accessibility were significantly enriched for terms relating to salt or osmotic stress response when compared to genes with just increased expression (Supplemental Table 4), suggesting that up-regulation of key genes in the response to osmotic stress is modulated through changes in chromatin architecture at the promoter.

We further analyzed promoters with increasing accessibility during the first 15 min of the time-course for accompanying shifts and/or depletion of nucleosomes. We observe that, for promoters with increased accessibility, the +1 nucleosome often exhibits significant downstream shifts relative to the TSS (median shift = 5 bp, *P* < 10^−5^ by bootstrap sampling) (Fig. 5B). In contrast, the position of the −1 nucleosome does not appear to systematically shift in either direction. However, the −1 nucleosome shows a systematic decrease in nucleosome occupancy in promoters exhibiting increased accessibility (median difference in occupancy = −0.09; *P* < 10^−5^ by bootstrap sampling) (Fig. 5C). While some decreases in occupancy for the +1 nucleosome can be observed, this effect is less pronounced (median difference in occupancy = −0.03; *P* < 10^−5^ by bootstrap sampling) (Fig. 5C). To explore these observations further, we classified genes as having a downstream shift in the +1 nucleosome (between 20 and 73 bp), depletion of the −1 nucleosome (occupancy change > 0.2), and/or depletion of the +1 nucleosome (Fig. 5D). Changes in positioning or depletion of the +1 nucleosome tend to occur in promoters already containing a nucleosome-free region, while depletion of the −1 nucleosome occurred for promoters both with and without a pre-existing nucleosome-free region (Fig. 5D).

**Figure 6.**
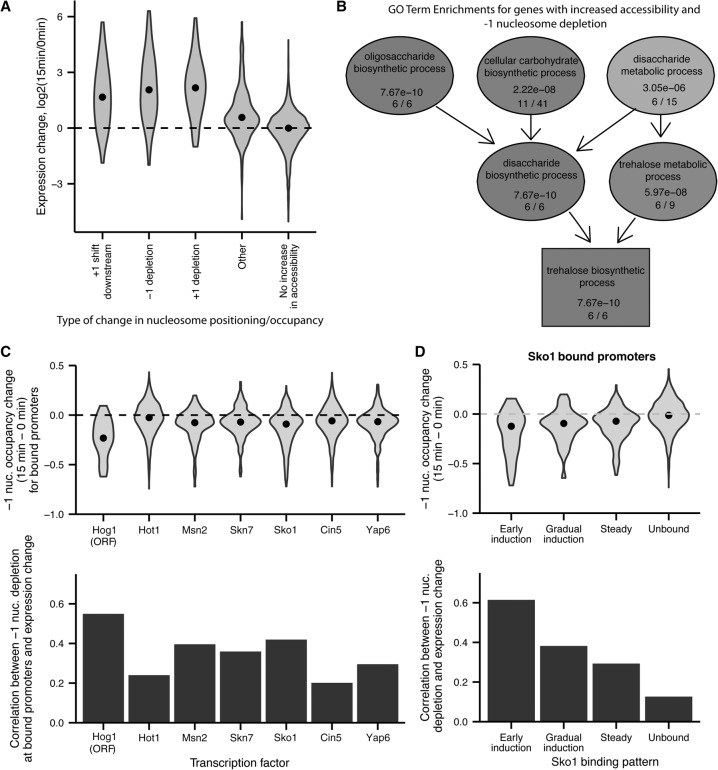
Changes in nucleosome positioning and occupancy during osmotic stress are linked to expression changes and mediated by TF binding. (*A*) Distribution of expression changes for promoters showing increased accessibility as well as different types of changes in nucleosome positioning or occupancy. (*B*) GO Term enrichment graph for genes with increased accessibility and depletion of the −1 nucleosome during the first 15 min of osmotic stress. (*C*) Distribution of changes in −1 nucleosome occupancy (*top*) and correlation between −1 nucleosome depletion and expression increases (*bottom*) for promoters bound by different TFs. (*D*) Distribution of changes in −1 nucleosome occupancy (*top*) and correlation between −1 nucleosome depletion and expression increases (*bottom*) for promoters with different Sko1 binding patterns.

Each of these three patterns of nucleosome changes is associated with expression increases greater than that observed for promoters that do not have any of these patterns (Fig. 6A). Genes with increased accessibility and −1 nucleosome depletion are highly enriched for the GO term “trehalose biosynthetic process,” with all six of the genes annotated with this term and included in our analysis being characterized by depletion of the −1 nucleosome and increasing accessibility (Fig. 6B; Supplemental Table 5). To determine what regulatory factors may be driving −1 nucleosome depletion, we determined the distribution of −1 nucleosome occupancy changes for promoters bound by a variety of different factors during osmotic stress as determined by previous ChIP studies (Fig. 6C; Ni et al. 2009; Cook and O'Shea 2012). Gene bodies bound by Hog1 are strongly depleted at the −1 nucleosome (median difference in occupancy [15–0 min] = −0.23; *P* < 10^−5^ by bootstrap sampling), as has been previously observed (Mas et al. 2009; Nadal-Ribelles et al. 2012), and this depletion of the −1 nucleosome is strongly correlated with increases in expression (*r* = 0.55, *P* = 0.005). The strongest association for a TF other than Hog1 is Sko1, a factor previously characterized as a master regulator of the osmotic stress response (Ni et al. 2009). To further explore the temporal relationship between Sko1 binding and −1 nucleosome depletion, we determined the distribution of −1 nucleosome depletion for promoters with different Sko1 binding patterns, previously determined via ChIP. The subset of Sko1 binding sites characterized by early induction of Sko1 binding shows much more pronounced nucleosome depletion than binding sites characterized by steady or gradual induction (*P* = 0.00034, bootstrap sampling 100,000 times), and the correlation between expression increase and −1 nucleosome depletion is also stronger (*P* = 0.00708, bootstrap sampling 100,000 times) (Fig. 6D). These results suggest the binding of Sko1 early in the response to osmotic stress may play a role in the loss of the −1 nucleosome and drive subsequent gene expression changes.

### Transcription factors and nucleosomes compete for binding to DNA

**Figure 7.**
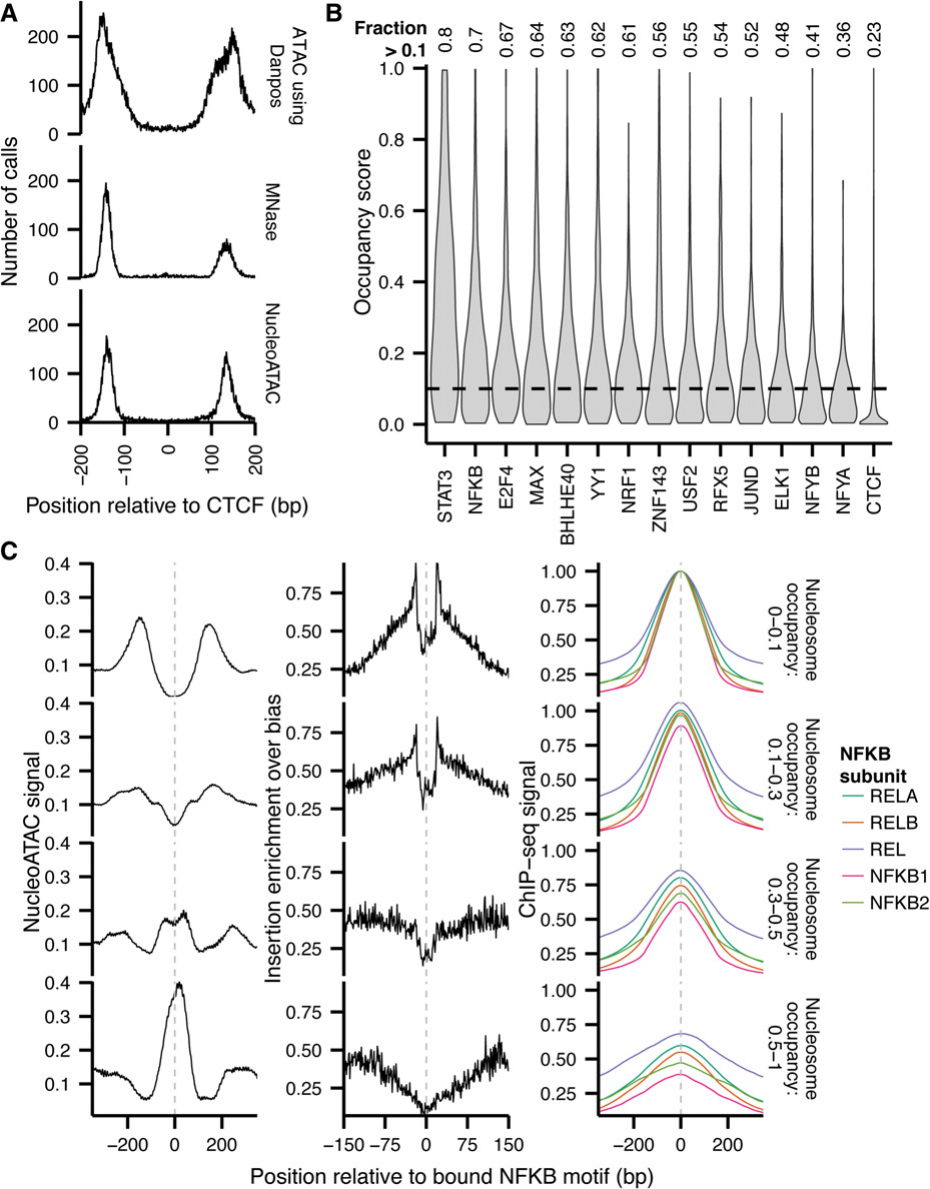
NucleoATAC defines stereotyped TF-nucleosome relationships. (*A*) Nucleosome dyad density relative to CTCF binding site for nucleosomes called previously with DANPOS (*top*), MNase (*middle*), or NucleoATAC (*bottom*). (*B*) Nucleosome occupancy distributions for sequence-specific TFs. (*C*) NucleoATAC nucleosome signal (*left*), ATAC-seq insertion profile (*middle*), and NFKB subunit ChIP-seq signal for NFKB at sites with different nucleosome occupancies (*right*). Insertion frequency normalized by sequence bias model. ChIP-seq intensities for each subunit were normalized such that the maximum intensity for the sites with 0 to 0.1 nucleosome occupancy was 1.

To demonstrate the ability of NucleoATAC to enable detailed investigation of the profiles of human nucleosomes around TFs, we assessed NucleoATAC calls around CTCF motifs. We observe high consistency in the distance of NucleoATAC calls from CTCF binding sites (Fig. 7A), concordant with previous observations that CTCF binding sites have highly stereotyped local nucleosome positioning (Fu et al. 2008; Buenrostro et al. 2013). Examining the distance to the nearest nucleosome for other sequence-specific transcription factors, we see that CTCF appears unique in its ability to strongly position flanking nucleosomes, as other TF binding sites often overlap with nucleosomes (Supplemental Fig. 18). To explore the relationship between nucleosome occupancy and TF binding more quantitatively, we determined the distribution of nucleosome occupancy for binding sites of 15 sequence-specific TFs (defined as motifs overlapping a ChIP-seq peak) (Fig. 7B). For most TFs other than CTCF, a substantial proportion of bound sites have a nonzero nucleosome occupancy score, although all show a preference for nucleosome-free DNA.

To further explore the relationship between nucleosomes and TF occupancy, we examined both the nucleosome signal and insertion pattern for NFKB sites with different nucleosome occupancy scores (Fig. 7C). Sites with very low nucleosome occupancy exhibited a clear depletion in the nucleosome signal and a clear transcription factor footprint (Hesselberth et al. 2009) shown by transposase insertion probabilities (i.e., characterized by a sharp drop in insertions within the motif site). In contrast, high nucleosome occupancy binding sites have a peak in nucleosome signal near the motif and a wide depletion of insertions, indicative of DNA protected by a nucleosome rather than a TF. In addition, sites with high nucleosome occupancy had lower ChIP-seq signal for five NFKB subunits (Zhao et al. 2014) than sites with low or intermediate nucleosome occupancy, showing that these sites are indeed less occupied by NFKB in aggregate. Notably, NFKB has been shown to dynamically oscillate between nuclear and cytoplasmic localization (Tay et al. 2010); this variability in localization may provide an explanation for observed intermediate levels of nucleosome occupancy—certain cells have a higher likelihood of being TF occupied vs. nucleosome occupied, depending on the nuclear concentration of the TF. All together, these results demonstrate that NucleoATAC may be used to infer dynamic competition between TFs and nucleosomes, with possible applications to understanding the molecular determinants of single-cell regulatory variability.

## Discussion

NucleoATAC utilizes the highly structured 2D fragment size versus midpoint “nucleosome fingerprint” from ATAC-seq chromatin accessibility data to generate high-resolution nucleosome maps within active regulatory elements. The 2D fingerprint derived from *S. cerevisiae* can be applied across species, assuming similar nucleosomal constraints on Tn5 insertion as suggested by the fragment-size distribution. These high-resolution maps are highly concordant to those identified by chemical cleavage in yeast and capture the rotational positioning information from nucleosomes in both yeast and humans. As expected, we observe that, for *S. cerevisiae* and *S. pombe*, WW (W = A or T) and SS (S = G or C) dinucleotides show strong 10- to 11-bp periodicity, but we also observe that all other dinucleotides exhibit considerable periodicity in-phase with the SS dinucleotides. We find human nucleosomes show periodicity in some dinucleotides but that the extent of periodicity is lower and phasing is less consistent than for both yeast species, suggesting that in vivo human nucleosome positions are much less constrained by their inherent sequence preference. We also observe a depletion of AT content immediately flanking nucleosomes called by NucleoATAC, in contrast to MNase-based studies that have observed an enrichment of AT content in these regions (Valouev et al. 2011; Gaffney et al. 2012). Together, these data validate the hypothesis that human nucleosomes are, in part, positioned by their underlying sequence context in vivo, although sequence preferences at the nucleosome boundaries may result from differing sequence biases of the Tn5 and MNase enzymes (Chung et al. 2010; Fan et al. 2010; Buenrostro et al. 2013). This observation highlights the need for orthogonal approaches to studying human nucleosome architecture.

Most methods for determining nucleosome positions measure nucleosome depletion only indirectly through a lack of (unnormalized) signal. In contrast, ATAC-seq simultaneously assays nucleosome depletion (through the presence of short fragments) and nucleosome positioning (from longer fragments). Combining measurements of chromatin accessibility, nucleosome positioning, and nucleosome occupancy allows an integrative analysis of chromatin architectural changes, as demonstrated by the observation of specific types of changes in nucleosome positioning and occupancy during the osmotic stress response in *S. cerevisiae*. In addition to identifying transient decreases in the occupancy of the −1 nucleosome that correlate with strong expression changes, we found that downstream shifts in the +1 nucleosomes were also associated with increased promoter accessibility and expression. As well as observing that Hog1 bound genes show strong −1 nucleosome depletion as has been previously characterized (Mas et al. 2009; Nadal-Ribelles et al. 2012), we show that a specific Sko1 binding pattern is strongly associated with −1 nucleosome depletion. These results highlight the ability of NucleoATAC to precisely interrogate changes in chromatin architecture during a dynamic process.

High-resolution nucleosome calls and occupancy tracks also enabled investigation of the effects of nucleosome occupancy on TF binding within regulatory regions. Some factors show intermediate nucleosome occupancy at a substantial fraction of binding sites, suggesting cell-to-cell heterogeneity in TF occupancy. This heterogeneity may be linked to oscillation in nuclear localization of TFs (Levine et al. 2013); examining which sites show partial occupancy may reveal new insight into how TF pulsing dynamics influence binding and gene regulation.

NucleoATAC provides a powerful new framework for analyzing ATAC-seq, MNase, and other paired-end functional genomics data. By using a two-dimensional fragment size versus midpoint representation of sequencing data, NucleoATAC is built on the understanding that different fragment lengths provide unique information content. Our 2D signal processing approach can likely be extended by applying additional methodologies from the image analysis field, opening exciting possibilities for future applications for calling genomic features other than nucleosomes.

ATAC-seq coupled with NucleoATAC allows for the interrogation of high-resolution nucleosome positions in regulatory regions from limited cellular populations, allowing rapid, cost-effective, and high-resolution nucleosome inference. We believe future efforts will include fine mapping of chromatin structure in rare developmental and disease cellular populations, providing a detailed understanding of the molecular determinants of chromatin structure across dynamic cellular processes in human cells.

## Methods

### Strains, library preparation, sequence processing, and peak calling

The GSY147 strain (Lee et al. 2008) was used for *S. cerevisiae*, except for the osmotic stress time-course, for which *S. cerevisiae* strain BY4741 was used. Strain 972 h- was used for *S. pombe*. *S. cerevisiae* and *S. pombe* samples were spheroplasted prior to incubation with Nextera Transposase; PCR was performed as previously described (Buenrostro et al. 2015). Bowtie 2 (Langmead and Salzberg 2012) was used to align *S. cerevisiae* reads to the sacCer3 genome (April 2011 Release from *Saccharomyces* Genome Database [Cherry et al. 2012]), *S. pombe* reads to the ASM294v2.21 genome, and GM12878 reads to the hg19 genome. For all species, open chromatin regions were called using MACS2 (Zhang et al. 2008) with the broad flag and were filtered based on mappability. For further details on library preparation, sequence processing, and peak-calling, see Supplemental Methods.

### Insertion position and fragment size determination

The start of sequencing reads generated from ATAC-seq are offset from the center of the Tn5 binding site by 4 bp (Buenrostro et al. 2013). Thus, ATAC-seq insertions were defined as single base-pair sites 4 bp from the ends of sequencing fragments. Similarly, fragment size was defined as the size of the sequenced fragment minus 8 bp so that fragment size represents the distance between the centers of two Tn5 binding sites.

### Occupancy determination

We sought to model the fragment-size distribution as a mixture of nucleosome-free fragments and nucleosome-associated fragments in a way that captured the highly structured, nonparametric nature of the nucleosomal distribution. Because fragments <115 bp very likely arise from the nucleosome-free distribution, we parameterized the fragment-size distribution below that size-cutoff as an exponential distribution, which provided a good fit to this region of the distribution. This fit distribution was used to extrapolate the nucleosome-free fragment distribution for sizes larger than 115 bp. The subtracted difference between the extrapolated nucleosome-free model and the observed fragment distribution was used as the nucleosome-associated fragment distribution. The fragment-size distribution was then modeled as a mixture of the nucleosomal and nucleosome-free insert size distributions:

$$P(i)=\alpha *P_{\mathrm{nucleosomal}}(i)+(1-\alpha )*P_{\mathrm{nucleosome}-\mathrm{free}}(i),$$

where α represents the fraction of fragments arising from the nucleosomal distribution and is bounded between 0 and 1 (inclusive). Nucleosome occupancy tracks were determined by computing the maximum likelihood estimate of α for fragments centered in 121-bp windows across the genomes at 5-bp intervals. This track was then smoothed using a 121-bp Gaussian window with a standard deviation of 20 bp. Confidence interval tracks were also computed for the occupancy track using the 90% confidence interval estimates for α for the same windows and performing the same smoothing.

### V-plot normalization

The yeast V-plot used for cross-correlation was generated by aggregating reads around dyad calls from chemical mapping that met two criteria: (1) They had an NCP/noise ratio (positioning metric defined previously [Brogaard et al. 2012]) in the top 20% of calls; and (2) they had nucleosomal occupancy (determined as described above) of >0.5. The portion of the V-plot representing fragments of sizes between 105 and 250 bp with fragment centers within 60 bp of the dyad position was normalized to match the nucleosomal fragment-size distribution of the sample being analyzed. For this normalization, we used the initial mixture model for the fragment-size distribution to determine a refined nucleosome-associated fragment distribution. Peaks in the nucleosome occupancy track—as determined from the initial model—were identified as candidate, low-resolution nucleosome positions. The fragment-size distribution for fragments centered within 60 bp of these peak positions was then used as the nucleosomal fragment size for the V-plot normalization. Each row in the V-plot corresponds to a specific fragment size; the elements in a particular row were all scaled so that the sum of the row would match the frequency of that fragment size in the nucleosome-associated fragment-size distribution. The V-plot was also symmetrized across the vertical axis and smoothed slightly with a Gaussian filter with a standard deviation of 1 bp.

### Nucleosome signal track and background subtraction

This V-plot was cross-correlated against matrices defining the fragment center and size information for a genomic region, such that the cross-correlation signal at position *x* along the genome is given by

Signal(x)=F⋅V

where *F* is the matrix of fragment center and size information for fragments of size 105 to 250 bp with centers between *x* − 60 and *x* + 60 and V is the V-plot matrix. This raw signal is then normalized using a background signal that is intended to represent the expected signal from the cross-correlation, given (1) the number of fragments observed, and (2) the Tn5 sequence preference. The background signal at position x is defined as

Background(x)=B⋅V∗∑F,

where *B* represents a matrix with relative probabilities of generating fragments of different sizes and center positions such that ∑*B* = 1. The scaling factor ∑*F*, the sum of all reads in the signal matrix, ensures that the background signal represents the expected signal given the observed number of fragments. To determine *B*, the probability of observing individual insertion sites was first modeled as follows. Tn5 has a sequence preference across ∼21 bp that it contacts (Buenrostro et al. 2013); therefore, we developed a Position Weight Matrix (PWM) for sequence content ±10 bp from Tn5 insertion points in ATAC-seq performed on genomic DNA. Relative probabilities are calculated for each genomic position using this PWM, and then this 1D sequence preference is used to calculate the relative probability of observing particular ATAC-seq fragments (which require two Tn5 insertions) by multiplying the probabilities of the two insertions needed for that fragment with the probability of observing a fragment of that size (determined from the fragment-size distribution). The normalized nucleosome signal is given by subtracting this background signal from the cross-correlation signal:

Normalized Signal(x)=F⋅V−B⋅V∗∑F.

### Calling dyad positions

The normalized nucleosome signal tends to be highly periodic with many local maxima. To robustly identify maxima representing potential nucleosome dyad positions while still preserving the rotational positioning information in the periodic signal, the normalized signal is smoothed using a Gaussian window of 25 bp, and local maxima are found in the sum of this smoothed signal and the original normalized signal. These local maxima are considered candidate nucleosome positions. To define a nonredundant map of nucleosome positions, a greedy algorithm is employed in which the candidate nucleosome position with the highest signal is chosen to be included in the map, then the next highest peak not within 120 bp of any position in the map already, until no peaks remain that are not within 120 bp of the nonredundant set.

For each dyad, a *Z*-score is determined by calculating

$$Z=(F\cdot V-B\cdot V*\sum F)/\sqrt{\text{var}(\text{Background})}$$

where the variance of the background signal based on the bias model is

$$\text{var}(\text{Background})=\sum F*\left(\sum b_{k}*(1-b_{k})*v_{k}^{2}-\underset{k!=l}{\sum }b_{k}*b_{l}*v_{k}*v_{l}\right),$$

with *b*_k_ and *b*_l_ as individual elements of matrix B and *v*_k_ and *v*_l_ the corresponding elements of matrix V. A log-likelihood ratio is also determined by calculating the likelihood of the data given that the data arises from the V-plot pattern multiplied by the local bias pattern and the likelihood of the data arising purely from the local bias pattern. For all analyses, only calls with *Z*-scores >3 (corresponding to a *P*-value of ∼0.001) and log-likelihood ratios >0 were considered. These cutoffs were chosen based on three factors: (1) analysis of ATAC insertion profile and V-plot around calls; (2) concordance between calls and chemical mapping; and (3) concordance between calls for individual replicates. As can be seen in Supplemental Figure 10, increasing the stringency of thresholds leads to more consistent calls that are more concordant with chemical mapping. The choice of thresholds for different applications should be based on the desired balance between more comprehensive calls and higher confidence calls.

### Positional concordance metrics

In order to assess the quality of a set of nucleosome position calls, we used several metrics that measure the concordance of the calls with a “gold standard” data set. The chemical mapping data sets from *S. cerevisiae* and *S. pombe* were used as the gold standard data sets. Concordance metrics were adapted from Mammana et al. (2013). The distance AUC was defined as the area under the curve for the cumulative distance plot for the distances between a call in the test data set and the nearest call in the gold standard data set within 73 bp (with test calls with no calls in the gold standard data set within 73 bp excluded). Specificity was defined as the fraction of calls for which the nearest call in the gold standard data set was within 25 bp. Sensitivity was defined as the faction of calls in the gold standard data set for which the nearest call in the test data set was within 25 bp. A “rotational specificity” metric was developed to measure how many of the nucleosome positions match a physiological nucleosome position; this metric is defined as the faction of calls for which the nearest call in the redundant chemical mapping data set (all nucleosome positions determined from chemical mapping without consideration of overlap) is within 1 bp.

### NucleoATAC applied to MNase

Several changes to the NucleoATAC workflow were made for application to MNase data. Nucleosome occupancy was not computed via the method outlined for ATAC-seq, as short fragments were removed via size-selection for both samples analyzed; rather the number of MNase fragments centered within 60 bp of a position was used as the occupancy. Additionally, a sequence bias model was not used, as modeling the sequence bias of a processive enzyme is not straightforward. A background model was still used; however, the model simply represents fragments positioned at random given the fragment-size distribution.

### Dinucleotide pattern analysis

For comparison of dinucleotide frequencies between species, a higher confidence threshold was used—only calls with log likelihood ratios ≥5 were used (threshold chosen based on analysis of random down-samples and individual replicates, as in Supplemental Figs. 8,9). For both correlation and power spectrum density analysis, dinucleotide frequencies between 19 and 60 bp from the dyad calls (averaged across both sides of the dyad, as the calls were not inherently stranded) were normalized by division with the mean frequency of the dinucleotide in that window. The power spectrum density at frequency 1/10.5 was calculated as the square of the fast Fourier transform at that frequency.

### Osmotic stress time-course analysis

For differential accessibility analysis between time points, we compared the number of insertions in promoters (−400 to +100 bp relative to TSS) between time points. As variation in the degree of enrichment of fragments within open chromatin regions can affect differential accessibility measurements between ATAC-seq samples, counts were normalized as follows: Quantile normalization was applied to the counts for promoters with genes showing no expression change as determined by a previous study (Ni et al. 2009). (Raw counts for these genes were highly correlated.) A lowess curve was then fitted to the quantile-normalized counts versus raw counts for each sample to map the smooth transform to be applied to the raw counts in that sample. This transform was then applied to all genes. The natural log of the difference between the normalized and raw counts was used as an offset in a GLM model regressing observed counts on the salt induction time point using the R package edgeR (Robinson et al. 2011). A likelihood ratio test was performed to identify gene that changed significantly between 0 and 15 min at an FDR of 0.01. The +1 and −1 nucleosome positions relative to TSS were determined using NucleoATAC signal and occupancy. For Figure 6C, we used ChIP-seq calls from Cook and O'Shea (2012) for Hog1 and Hot1 and ChIP-seq calls from Ni et al. (2009) for all other TFs.

### Downloaded data sets and annotations

Chemical mapping data for *S. cerevisiae* were obtained from Supplemental Table 2 from Brogaard et al. (2012) and lifted over to the sacCer3 genome. Chemical mapping for *S. pombe* were obtained from Supplemental Data Set 01 from Moyle-Heyrman et al. (2013). MNase data (Cole et al. 2011) used for Figure 2A,B and Table 1 were obtained from SRA (SRR094649.sra and SRR094650.sra). A second MNase data set (Gossett and Lieb 2012) (used for Supplemental Table 2 and Supplemental Fig. 10) was obtained from SRA (SRR208072.sra, SRR208073.sra, and SRR208075.sra). For both data sets, FASTQ files were aligned to the sacCer3 genome using Bowtie 2 and filtered for reads with mapping quality ≥30. For calling nucleosomes with MNase using DANPOS2 (Chen et al. 2013), default parameters were used except that the --paired flag was set to 1. For calling nucleosomes with MNase using PuFFIN (Polishko et al. 2014), default parameters were used. Human (GM) MNase fragment center positions mapped to hg19 were obtained from the Pritchard laboratory by request. Positioned human nucleosomes called by MNase for GM cells were downloaded from http://eqtl.uchicago.edu/nucleosomes/positioning_scores/peaks.min_peak_score_0.6.thresh_0.5.txt.gz and lifted over from hg18 to hg19.

For *S. cerevisiae*, TSSs were determined using median UTR lengths from TIF-Seq (Pelechano et al. 2013) and gene annotations from the *Saccharomyces* Genome Database (Cherry et al. 2012). *For S. pombe*, TSSs were obtained from Supplemental Table 2 from Lantermann et al. (2010). For human, TSSs were defined by CAGE signal from the ENCODE Project Consortium (The ENCODE Project Consortium 2012); for each transcript, only the TSS with maximum CAGE signal was used. For Figure 6, uniformly processed ENCODE/SYDH ChIP-seq data sets were downloaded from the UCSC ENCODE data repository (http://hgdownload.cse.ucsc.edu/goldenPath/hg19/encodeDCC/wgEncodeAwgTfbsUniform/). ChIP-Seq peaks were intersected with motif occurrences called using FIMO (Grant et al. 2011) and the JASPAR database (Sandelin 2004).

ATAC-seq data for GM12878 are available at the NCBI Gene Expression Omnibus (GEO; http://www.ncbi.nlm.nih.gov/geo/) under accession number GSM1155960 (Buenrostro et al. 2013).

## Data access

The raw data for *S. cerevisiae* and *S. pombe* as well as nucleosome positions and signal tracks for all three species analyzed have been submitted to the NCBI Gene Expression Omnibus (GEO; http://www.ncbi.nlm.nih.gov/geo/) under accession number GSE66386. NucleoATAC source code is freely available as a python package at https://github.com/GreenleafLab/NucleoATAC, as well as in the Supplemental Material.

## Competing interest statement

J.D.B. and W.J.G. are listed as inventors on a patent for the ATAC-seq method. W.J.G. is a scientific cofounder of Epinomics.

## Supplementary Material

Supplemental Material
