# Supplementary material for: Structured nucleosome fingerprints enable high-resolution mapping of chromatin architecture within regulatory regions
Source: Genome Res. 2015 Nov;25(11):1757–70. doi: 10.1101/gr.192294.115 (PMC4617971; doi:10.1101/gr.192294.115)
Supplement: Supplemental Material [file supp_25_11_1757__index.html]

Structured nucleosome fingerprints enable high-resolution mapping of chromatin architecture within regulatory regions — Structured nucleosome fingerprints enable high-resolution mapping of chromatin architecture within regulatory regions — Supplemental Material 

# Structured nucleosome fingerprints enable high-resolution mapping of chromatin architecture within regulatory regions

## Supplemental Material

**Files in this Data Supplement:**

- Supplemental Material.pdf
- NucleoATAC-software.zip
