# Supplementary material for: Structured nucleosome fingerprints enable high-resolution mapping of chromatin architecture within regulatory regions
Source: Genome Res. 2015 Nov;25(11):1757–70. doi: 10.1101/gr.192294.115 (PMC4617971; doi:10.1101/gr.192294.115)
Supplement: Supplemental Material [file supp_gr.192294.115_Supplemental_Material.pdf]

Supplementary Materials

for

Structured nucleosome fingerprints enable high-resolution mapping of chromatin architecture within regulatory regions

Alicia N. Schep[1], Jason D. Buenrostro[1], Sarah K. Denny[2], Katja Schwartz[1], Gavin Sherlock[1], William J. Greenleaf[1*]

[1]Department of Genetics, Stanford University School of Medicine, Stanford, CA 94305, USA

[2]Biophysics Program, Stanford University School of Medicine, Stanford, CA 94305, USA

*To whom correspondence should be addressed. E-mail: wjg@stanford.edu

Supplementary Methods

Yeast strains, growth, and library preparation

Four populations of *S. cerevisiae* strain GSY147 (Lee et al. 2008) were used, with three of the four populations derived from the first after being successively streaked on plates for approximately 200 generations in conditions to minimize selective pressures. Replicates were grown to mid-log phase in YPD, then 5 million cells per library were harvested. The cells were washed twice in Sorbitol buffer (1.4 M Sorbitol, 40 mM HEPES-KOH pH 7.5, 0.5 mM $MgCl_2$) then incubated for 30 min at 30°C shaking at 300 rpm with 0.5 mg/mL 100T zymolyase. The cells were washed twice in Sorbitol Buffer, then incubated with 2.5 µl of Nextera Transposase in 47.5 µl of 1x TD buffer at 37°C for 30 minutes. PCR was performed as previously described (Buenrostro et al. 2015). The libraries were sequenced with 50bp paired-end reads on an Illumina HiSeq.

For the time course analysis of osmotic stress, *S. cerevisiae* strain BY4741 was grown in YPD until 0, 15, 30, 45, or 60 minutes before harvesting, at which point a 5M NaCl solution in water was added for a final concentration of 0.6 M NaCl. Two replicates were used for each time point, except for time 0 for which there were four replicates. For all time points other than two of the replicates for time 0, Sorbitol Buffer washes and incubations prior to transposition were carried out in buffer with 0.6M NaCl. To accelerate the time between harvesting the cells and performing the transposition, the protocol was modified as follows: Cells were washed only once in Sorbitol buffer with 10mM DTT before being incubated for 5 minutes at 30°C and shaking at 300 rpm with 0.5 mg/mL 100T zymolyase in Sorbitol Buffer with 10mM DTT. Cells were then washed once with Sorbitol buffer before being incubated with 2.5 l of Nextera Transposase in 47.5 l of 1x TD buffer at 37°C and shaking at 300 rpm for 15 minutes. PCR was performed as previously described (Buenrostro et al. 2015). Libraries were sequenced with 76 bp paired-end reads on an Illumina NextSeq 500.

S. pombe strain 972 h- was grown to mid-log phase in YES media, and 1, 5, or 20 million cells were washed twice in Sorbitol Buffer + 10 mm BME, incubated in Sorbitol Buffer + 2mM BME for 30 minutes, and the incubated in Sorbitol buffer with 0.25 mg/mL 100T Zymolyase for 5 or 23 minutes, and then washed twice in Sorbitol Buffer before incubated in 10 µl of 1x TD buffer and 0.5 µl of Nextera Transposase at 37°C for 30 minutes. PCR was performed as previously (Buenrostro et al. 2015). Libraries were sequenced with 76 bp paired-end reads on an Illumina MiSeq.

Sequencing alignment and peak calling

Bowtie 2 (Langmead and Salzberg 2012) was used to align *S. cerevisiae* reads to the sacCer3 genome (April 2011 Release from *Saccharomyces* Genome Database (Cherry et al. 2012)), *S. pombe* reads to the ASM294v2.21 genome, and GM12878 reads to the hg19 genome. Duplicates were removed for each individual library using Picard tools (http://broadinstitute.github.io/picard). Reads with mapping quality below Q30 as well as improperly paired reads were removed from subsequent analysis. For the 11 *S. cerevisiae* samples that were not part of the osmotic time course, replicates across all biological samples were merged for all downstream analysis (unless otherwise specified). The 5 *S. pombe* replicates, 4 GM12878, and the 2 *S. cerevisiae* samples for each time point in the osmotic stress time-course were also merged for nucleosome analysis.

Determining broad open chromatin regions

For all species, open chromatin regions were called using MACS2 (Zhang et al. 2008) with the broad flag. Peaks were filtered based on mappability of 35 bp sequences; peaks with any 200 bp window with less than 75% of base-pairs mappable were removed (12% of S. cerevisiae, 9% of S. pombe, and 25% of GM12878). While many ATAC-seq fragments are longer than 35 bp, for the purposes of this analysis we wanted to remove the possibility of greater mappability of longer fragments skewing our nucleosome occupancy analysis. For the human data, the Duke 35 bp alignability track for hg19 was used to determine the mappability of peaks. For the two yeast species, mappability was determined by aligning all possible 35 bp reads from the genome using Bowtie 2 and determining which reads failed to align with high mapping quality.

Supplementary Note 1: Biases in nucleosome occupancy tracks

The nucleosome occupancy calculation performed by NucleoATAC attempts to ascertain the nucleosome occupancy within accessible genomic loci. Several systematic biases may influence this occupancy metric, which we discuss in turn: (1) Biases due to increased fragments from nucleosome free DNA. In general, more fragments are observed from identically sized regions that are nucleosome free rather than nucleosome occupied. Thus if at one locus the nucleosome occupancy is in reality 50%, the proportion of fragments arising from the loci that are nucleosomal rather nucleosome free is likely less than 50% and our nucleosome occupancy metric would underestimate the true nucleosome occupancy. (2) Biases due to background. Fragments arising from any contamination of free DNA or dead cells are likely to have a fragment size distribution more closely reflecting the nucleosome-free distribution than the nucleosomal distribution(Adey et al. 2010; Buenrostro et al. 2015). Thus confidence in regions of low occupancy increases with relative read density. (3) Biases due to assaying only accessible states. If there exists a heterogeneous population of cells in which a particular locus is accessible in a subset of cells and inaccessible in another subset, ATAC-seq will primarily assay the fraction that is accessible. The nucleosome occupancy determined from ATAC-seq should thus be interpreted as the occupancy within the accessible fraction, and may not be fully representative of the total nucleosome occupancy in the population.

Supplementary Note 2: Nucleosome positions missed by NucleoATAC & ATAC-seq

Even within the broad open chromatin regions considered for this study, NucleoATAC (using ATAC-seq) calls several thousand fewer nucleosomes than either chemical mapping or MNase. Outside of these broad open chromatin regions, the relative proportion of calls made by NucleoATAC is even lower. The primary reason for this lower sensitivity for calling nucleosomes is that ATAC-seq coverage is highly uneven across the genome, unlike MNase-seq or chemical mapping coverage. In less accessible chromatin, the use of ATAC-seq for nucleosome mapping is limited not only due to stochastic sampling noise but also due to lower signal-to-noise ratio. In ATAC-seq, noise can result from transposition into free genomic DNA or dead cells, in which the distribution of ATAC-seq fragment sizes and coverage is similar to that in genomic DNA(Adey et al. 2010; Buenrostro et al. 2015). Poor data for nucleosome mapping outside of open chromatin regions is a limitation of ATAC-seq and not specific to our NucleoATAC analysis method. Importantly, with ATAC-seq we can distinguish between regions with no nucleosome calls due to inaccessibility and regions with no nucleosome calls due to nucleosome depletion by considering the total fragment coverage at those regions. True regions of nucleosome depletion can be identified by the presence of relatively high coverage. If information about nucleosome positioning genome-wide is desired, the uneven coverage and poor signal to noise outside of open chromatin regions with ATAC-seq is problematic. However, the uneven coverage can be beneficial if one is interested in examining the positioning of nucleosomes that flank nucleosome-free regions.

By examining the ATAC-seq signal around calls for which MNase and chemical mapping agree but that are missed by NucleoATAC, we observe that nucleosome calls missed by NucleoATAC generally are characterized by lower ATAC-seq coverage than calls made by NucleoATAC. As the number of nucleosome calls made by NucleoATAC plateaus well below the total sequencing depth (**Supplementary Fig. 8-9**), the ability to make these calls is not limited by sequencing depth but rather signal-to-noise. The poor signal-to-noise can also be seen in the V-plot for missed calls with

relatively low ATAC-seq coverage; a weak nucleosome signature is present but the ratio of those fragments to shorter fragments is low (**Supplementary Fig. 6b**). For missed calls with higher coverage, the V-plot appears characteristic of nucleosome-depleted regions, with a strong enrichment of short fragments right at the position of the nucleosome call (**Supplementary Fig. 6c**). DNAse-seq cut density is enriched and MNase coverage is depleted at these nucleosome positions (**Supplementary Fig. 6de**), supporting the conclusion that these nucleosome calls fall within nucleosome-depleted area.

While for the *S. cerevisiae* data further sequencing would be unlikely to allow us to capture more nucleosomes, for the human data the number of nucleosomes called has not yet plateaued (**Supplementary Fig. 8**) and thus more nucleosomes than presently called are likely to be "visible" to ATAC-seq.

When applied to MNase data, NucleoATAC calls fewer nucleosomes than other analysis methods. However, the lower number of calls for MNase is due to stricter thresholds. By analyzing the Distance AUC versus number of calls at different thresholds, we observe that the distance AUC is superior for NucleoATAC at equal number of calls across different thresholds (**Supplementary Fig. 10d**). Loosening thresholds increases the number of calls made. As NucleoATAC was designed for ATAC-seq data, strict thresholds at the expense of comprehensive calls was considered important as for many areas of the genome ATAC-seq will simply not have the power to call positioned nucleosomes. With ATAC-seq and NucleoATAC, the absence of calls alone should not be interpreted as absence of nucleosome; coverage by many short fragments can be used to determine nucleosome-depleted regions.

Supplementary Table 1. Positional concordance metrics for nucleosome calls made using DANPOS2 with ATAC-seq. The ranges of fragment sizes used as the primary input to the DANPOS2 dpos tool were varied. Additionally, one analysis was performed without adding a "control" input of short, nucleosome-free fragments.

Fragment size range	Control fragment size range	Number of calls	Distance AUC	Sensitivity	Specificity	Rotational Specificity
116-250	0-115	14590	0.679	0.433	0.504	0.146
140-250	0-115	14261	0.685	0.436	0.521	0.149
140-250	None	17872	0.668	0.496	0.472	0.135
160-250	0-115	14172	0.680	0.432	0.519	0.139

Supplementary Table 2. Positional concordance metrics for second MNase data set (Gossett and Lieb 2012) using different analysis methods.

Assay	Inference method	Number of calls	Distance AUC	Sensitivity	Specificity	Rotational Specificity
MNase	NucleoATAC	14499	0.769	0.570	0.669	0.285
MNase	DANPOS2	18217	0.718	0.591	0.552	0.165
MNase	PuFFIN	17042	0.740	0.596	0.595	0.181

Supplementary Table 3. The most enriched biological process GO Terms for genes with increased promoter accessibility in first 15 minutes of osmotic stress. The background set is all genes considered in the analysis. Table shows all biological process GO terms with P-value < 1 x 10^{-4}.

GO ID	Term	Annotated	Significant	Expected	P-value
**GO:0055114**	oxidation-reduction process	338	65	36.1	6.5e-07
**GO:0009651**	response to salt stress	27	13	2.88	9.7e-07
**GO:0009628**	response to abiotic stimulus	125	32	13.35	1.3e-06
**GO:0005992**	trehalose biosynthetic process	6	6	0.64	1.4e-06
**GO:0009312**	oligosaccharide biosynthetic process	6	6	0.64	1.4e-06
**GO:0046351**	disaccharide biosynthetic process	6	6	0.64	1.4e-06
**GO:0006979**	response to oxidative stress	87	25	9.29	2.0e-06
**GO:0005996**	monosaccharide metabolic process	74	22	7.9	4.6e-06
**GO:0034599**	cellular response to oxidative stress	82	23	8.76	8.2e-06
**GO:0071214**	cellular response to abiotic stimulus	43	15	4.59	1.9e-05
**GO:0019318**	hexose metabolic process	69	20	7.37	1.9e-05
**GO:0044724**	single-organism carbohydrate catabolic process	64	19	6.83	2.1e-05
**GO:0006970**	response to osmotic stress	70	20	7.48	2.4e-05
**GO:0005991**	trehalose metabolic process	9	6	0.96	9.1e-05
**GO:0016052**	carbohydrate catabolic process	71	19	7.58	1e-04

Supplementary Table 4. The most enriched biological process GO Terms for genes with increased promoter accessibility and increased expression in first 15 minutes of osmotic stress relative to genes with increased expression. Table shows all biological process GO terms with P-value < 1 x 10^{-4}.

GO ID	Term	Annotated	Significant	Expected	P-value
**GO:0071214**	cellular response to abiotic stimulus	15	12	3.75	1.0e-05
**GO:0046496**	nicotinamide nucleotide metabolic process	17	12	4.25	8.4e-05
**GO:0009651**	response to salt stress	9	8	2.25	9.7e-05

Supplementary Table 5. The most enriched biological process GO Terms for genes with increased promoter accessibility and -1 nucleosome depletion in the first 15 minutes of osmotic stress. Background set is all genes considered in the analysis. Table shows all biological process GO terms with P-value < 1 x 10^{-4}.

GO ID	Term	Annotated	Significant	Expected	P-value
**GO:0005992**	trehalose biosynthetic process	6	6	0.19	7.7e-10
**GO:0009312**	oligosaccharide biosynthetic process	6	6	0.19	7.7e-10
**GO:0046351**	disaccharide biosynthetic process	6	6	0.19	7.7e-10
**GO:0016051**	carbohydrate biosynthetic process	65	13	2.01	5.1e-08
**GO:0044723**	single-organism carbohydrate metabolic process	210	23	6.48	5.6e-08
**GO:0005991**	trehalose metabolic process	9	6	0.28	6.0e-08
**GO:0044262**	cellular carbohydrate metabolic process	123	17	3.8	1.3e-07
**GO:0005975**	carbohydrate metabolic process	245	24	7.57	2.4e-07
**GO:0044724**	single-organism carbohydrate catabolic process	64	12	1.98	3.7e-07
**GO:0006112**	energy reserve metabolic process	25	8	0.77	4.6e-07
**GO:0016052**	carbohydrate catabolic process	71	12	2.19	1.2e-06
**GO:0005978**	glycogen biosynthetic process	14	6	0.43	1.9e-06
**GO:0005984**	disaccharide metabolic process	15	6	0.46	3.1e-06
**GO:0005977**	glycogen metabolic process	24	7	0.74	5.0e-06
**GO:0005996**	monosaccharide metabolic process	74	11	2.29	1.2e-05
**GO:0009250**	glucan biosynthetic process	19	6	0.59	1.5e-05
**GO:0006006**	glucose metabolic process	57	9	1.76	4.9e-05
**GO:0006073**	cellular glucan metabolic process	33	7	1.02	4.9e-05
**GO:0044042**	glucan metabolic process	33	7	1.02	4.9e-05
**GO:0006091**	generation of precursor metabolites and energy	137	14	4.23	6.4e-05
**GO:0009311**	oligosaccharide metabolic process	24	6	0.74	6.5e-05
**GO:0009628**	response to abiotic stimulus	125	13	3.86	9.9e-05

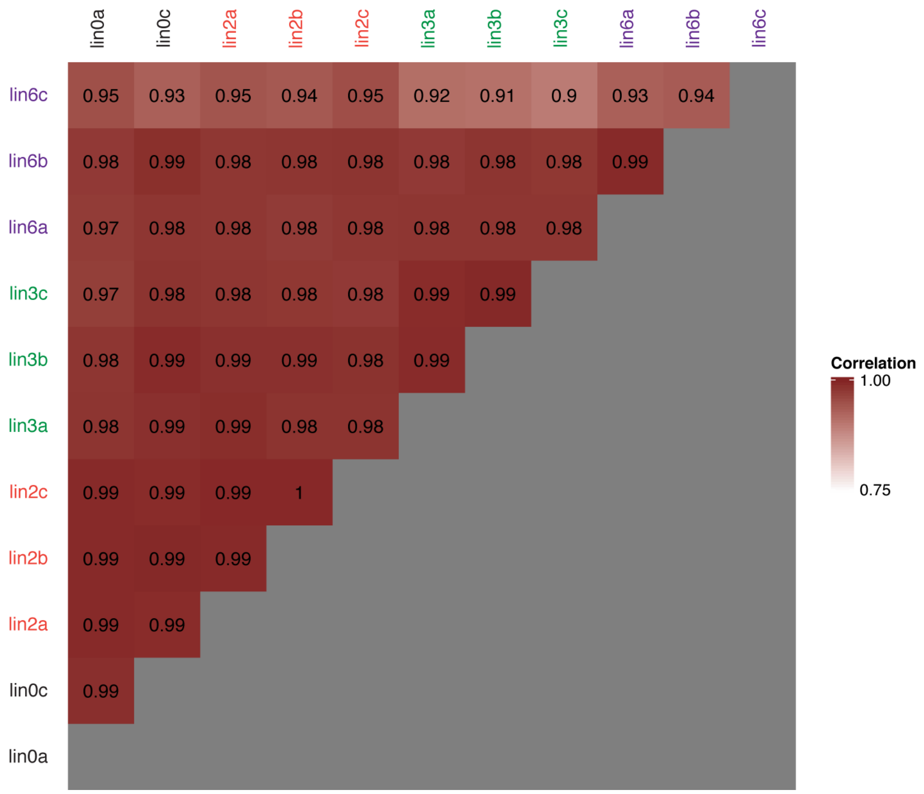

Supplementary Figure 1. Technical and biological replicates show highly correlated insertion patterns. Sets of technical replicates are shown in the same text color. Matrix shows the Pearson Correlation coefficient between the number of insertions in 50 bp windows across the genome for each pair-wise combination of samples. Correlation between replicates is greater than 0.89 for all comparisons.

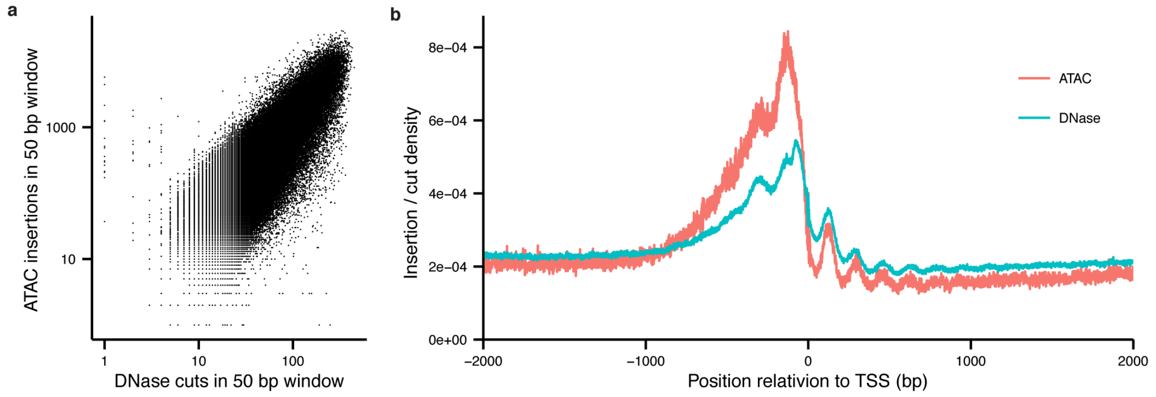

Supplementary Figure 2. High concordance between ATAC and DNase data. A) Number of ATAC-seq insertions versus DNase-seq cuts within 50bp windows across the *S. cerevisiae* genome. B) Profile of ATAC-seq insertions and DNase-seq cuts around transcription start sites in *S. cerevisiae*. Area under each curve is normalized to 1.

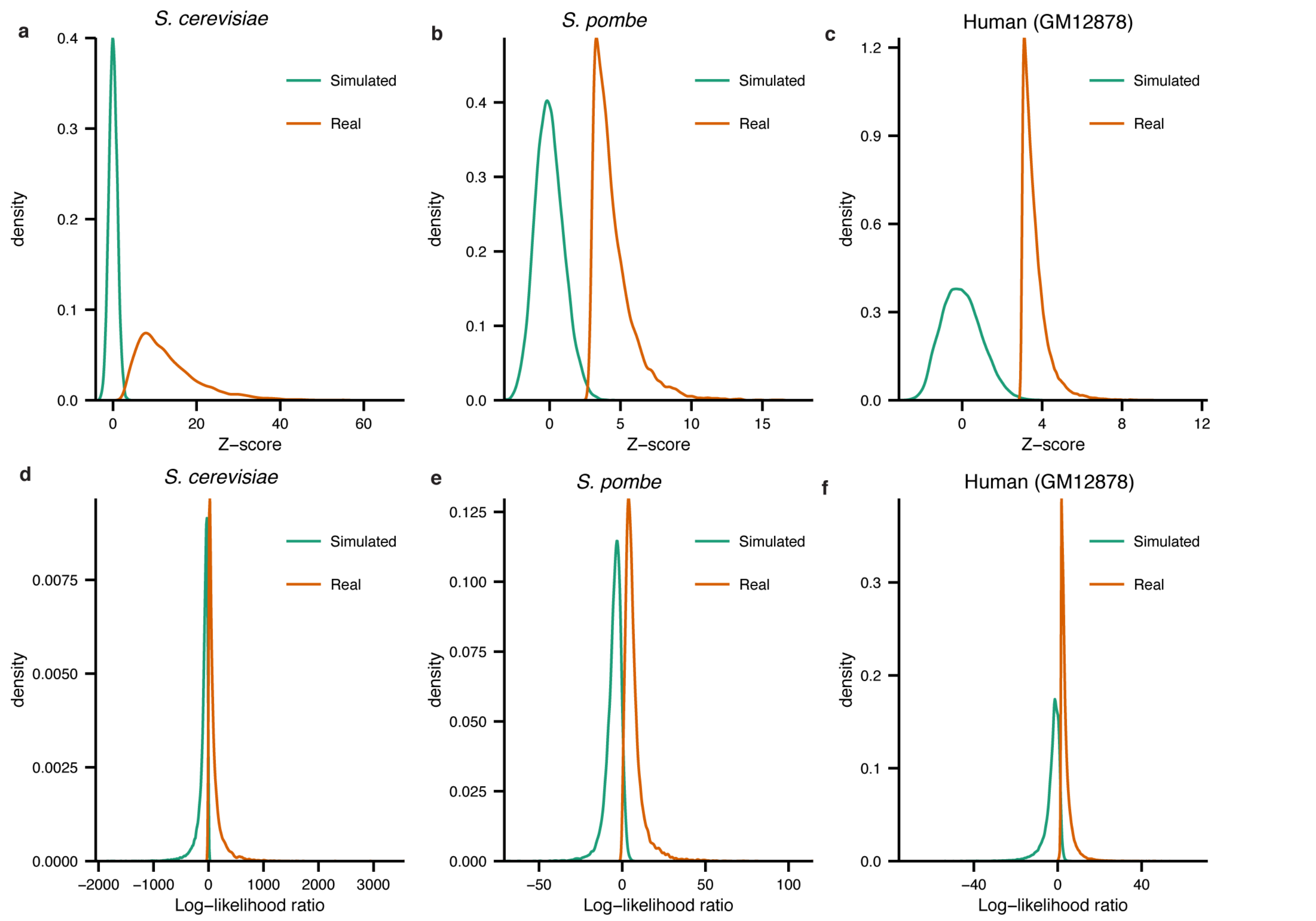

Supplementary Figure 3. Null distributions for Z-score and log-likelihood ratio. A) Distribution of Z-score at NucleoATAC calls when using either simulated data or real data for *S. cerevisiae*. B) Distribution of Z-score at NucleoATAC calls when using either simulated data or real data for *S. pombe*. C) Distribution of Z-score at NucleoATAC calls when using either simulated data or real data for GM12878 cells. D) Distribution of log-likelihood ratio at NucleoATAC calls when using either simulated data or real data for *S. cerevisiae*. E) Distribution of log-likelihood ratio at NucleoATAC calls when using either simulated data or real data for *S. pombe*. F) Distribution of log-likelihood ratio at NucleoATAC calls when using either simulated data or real data for GM12878 cells.

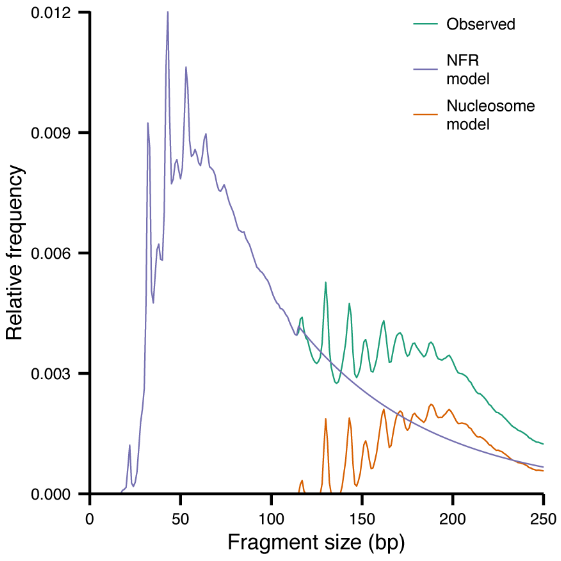

Supplementary Figure 4. Fragment size distribution is composed of nucleosome and nucleosome-free distributions. The nucleosome-free (NFR) model is determined by fitting an exponential curve to the observed distribution of sizes below 100 bp and then extrapolating for larger fragment sizes. The nucleosome distribution is determined simply by subtracting the NFR model from the observed distribution.

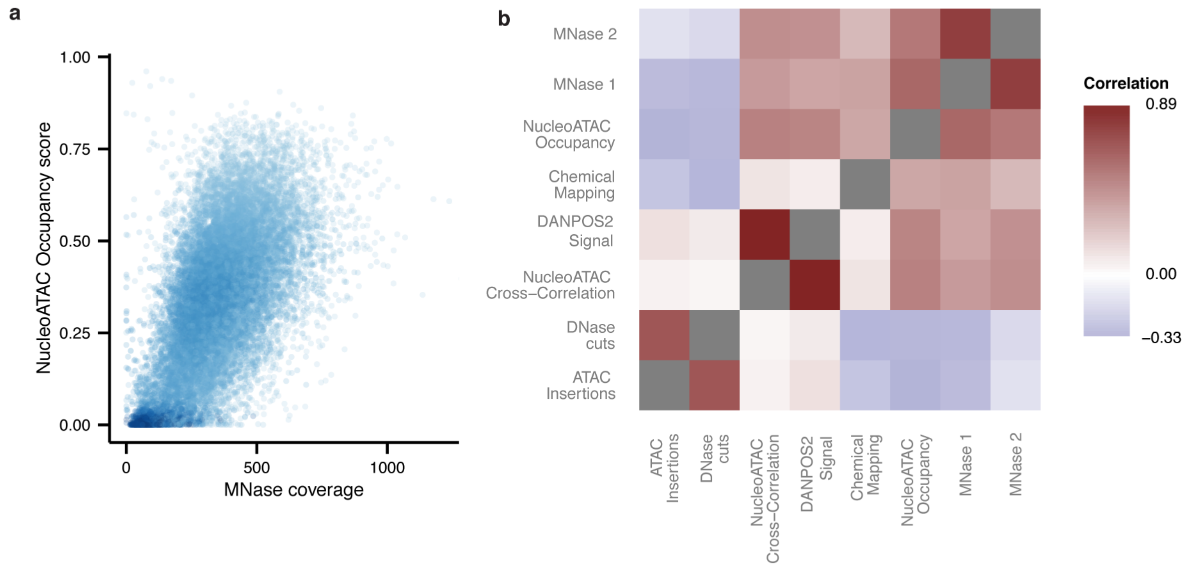

Supplementary Figure 5. Comparison between different nucleosome occupancy measurements. A) NucleoATAC occupancy score versus MNase coverage within 60 bp of chemical mapping nucleosome calls. B) Correlations between different types of nucleosome occupancy measurements. Nucleosome occupancies are at the positions of nucleosome calls determined by chemical mapping. ATAC insertions is the number of insertions within 60 bp. DNase cuts is the number of DNase cuts within 60 bp. NucleoATAC Cross-Correlation is the normalized cross-correlation signal. DANPOS2 signal is the background subtracted signal track created by DANPOS2 when using 140-250 bp fragments. Chemical mapping is nucleosome occupancy determined via chemical mapping as described in Moyle-Herriman et al. (2013). NucleoATAC Occupancy is the occupancy score computed from NucleoATAC. MNase1 is the number of MNase fragments from Cole et al. (2011). Centered within 60 bp MNase 2 is the number of MNase fragments from Gosset et al. (2012) centered within 60 bp.

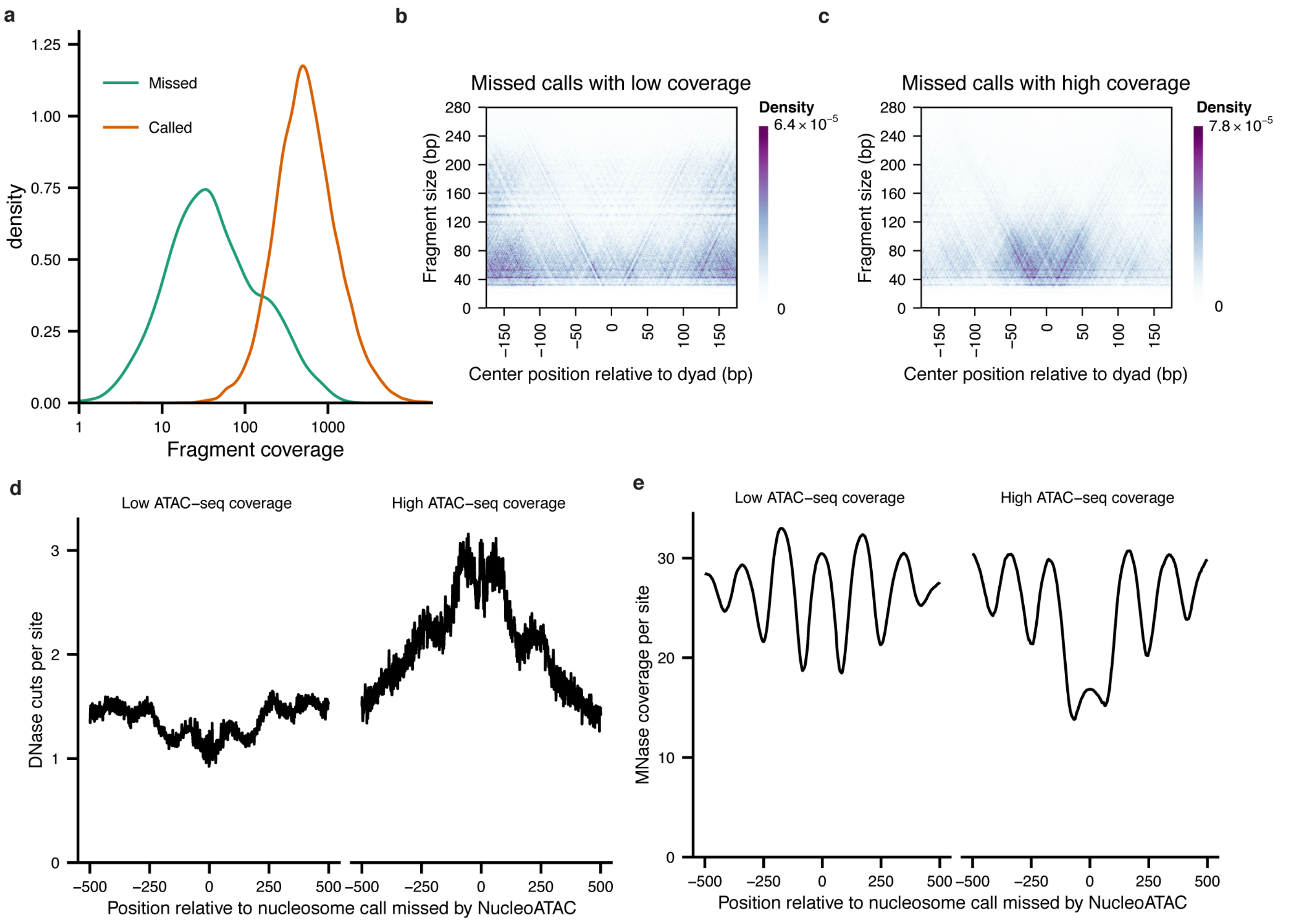

Supplementary Figure 6. Characteristics of nucleosomes missed by NucleoATAC. A) Distribution of coverage by fragments 0-250 bp in length for chemical mapping calls supported by MNase that are missed by NucleoATAC (green) and NucleoATAC calls (orange). B) V-plot for calls missed by NucleoATAC with low coverage (<100 fragments). C) V-plot for calls missed by NucleoATAC with high coverage (>100 fragments). D) DNase-seq signal around nucleosome calls missed by NucleoATAC with either low ATAC-seq coverage (left) or high ATAC-seq coverage (right). E) MNase-seq coverage around nucleosome calls missed by NucleoATAC with either low coverage (left) or high coverage (right). MNase coverage is fragment centers within 60 bp.

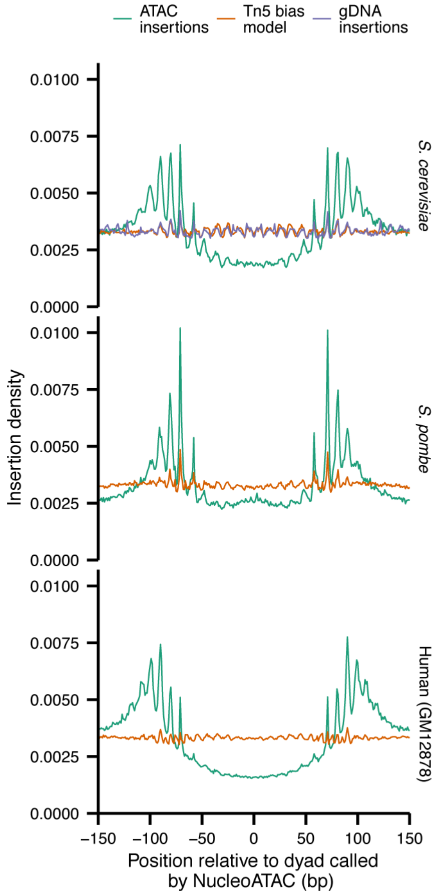

Supplementary Figure 7. Sequence bias is minimal around NucleoATAC calls. ATAC-seq insertion density (green), Tn5 bias model (purple), and Nextera genomic DNA (orange) insertions around NucleoATAC calls.

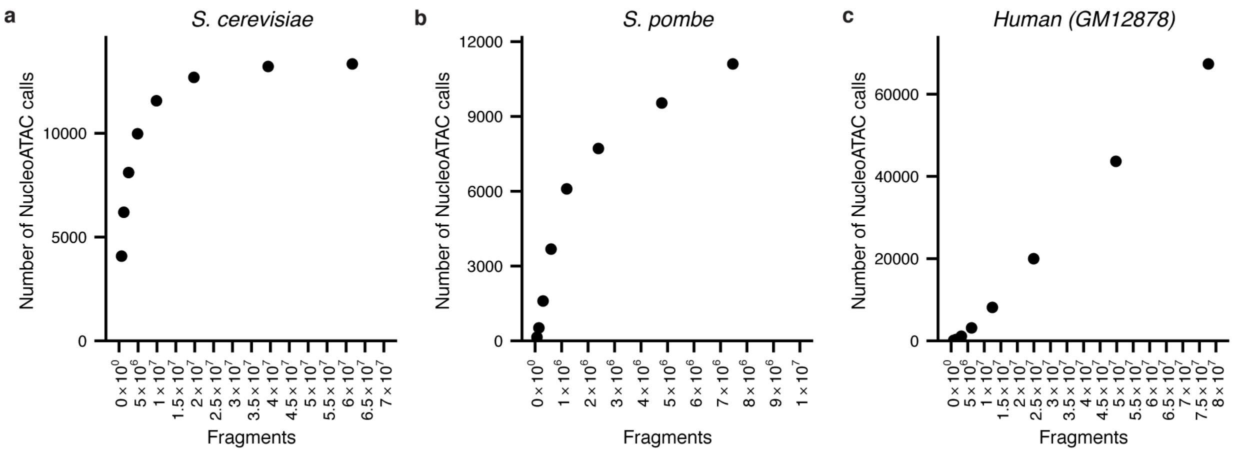

Supplementary Figure 8. Number of NucleoATAC nucleosome calls made versus number of sequencing fragments. The number of fragments represents only fragments that passed all filters as detailed in Methods section. A) *S. cerevisiae*, B) *S. pombe*, C) Human (GM12878 cells)

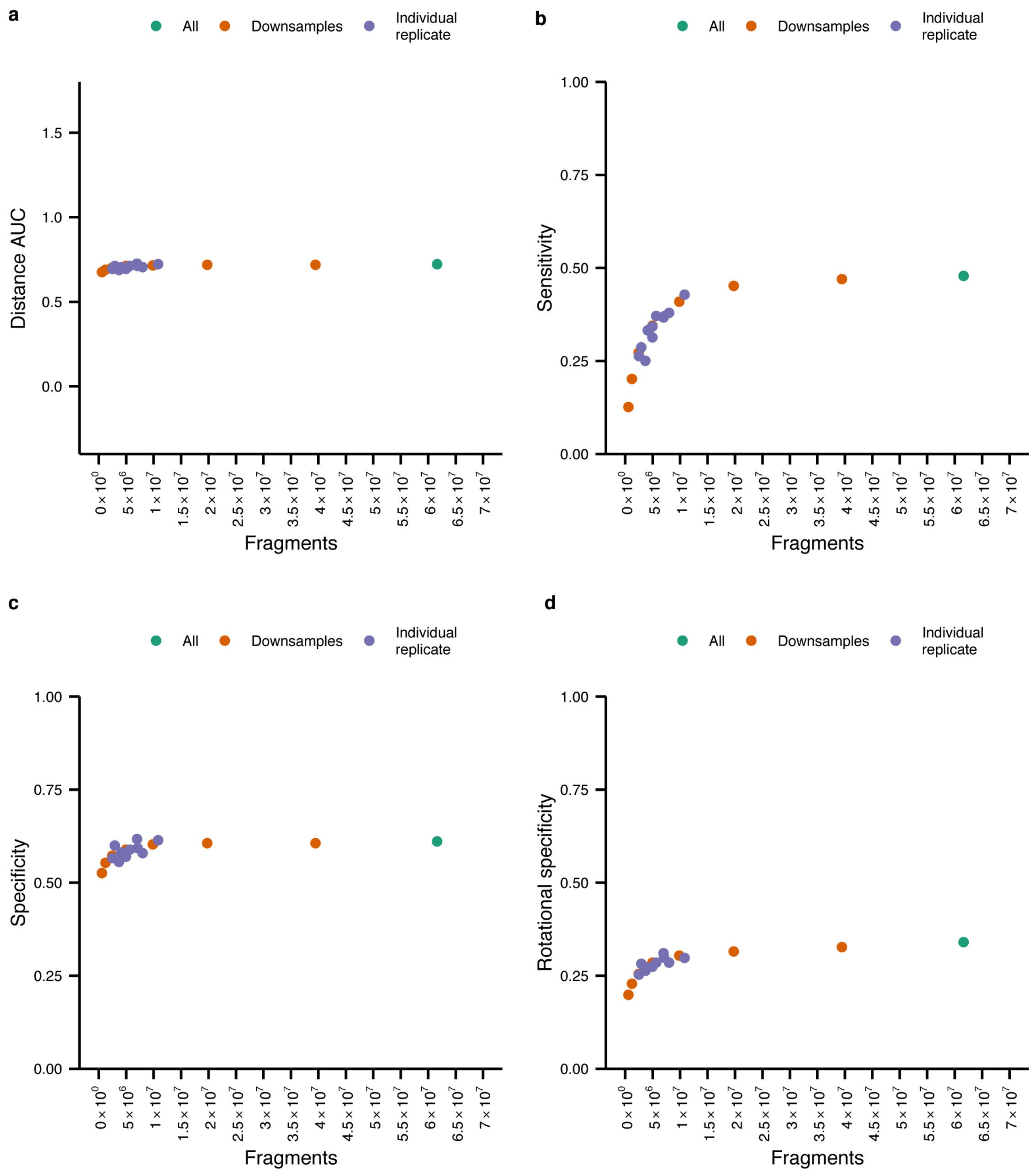

Supplementary Figure 9. Sequencing depth dependence of metrics for positional concordance with chemical mapping calls for *S. cerevisiae*. For all panels, the x-axis shows the number of fragments that passed all filters (mapping quality, no chrM) A) Distance AUC versus number of fragments. B) Sensitivity versus number of fragments. C) Specificity versus number of fragments. D) Rotational specificity versus number of fragments

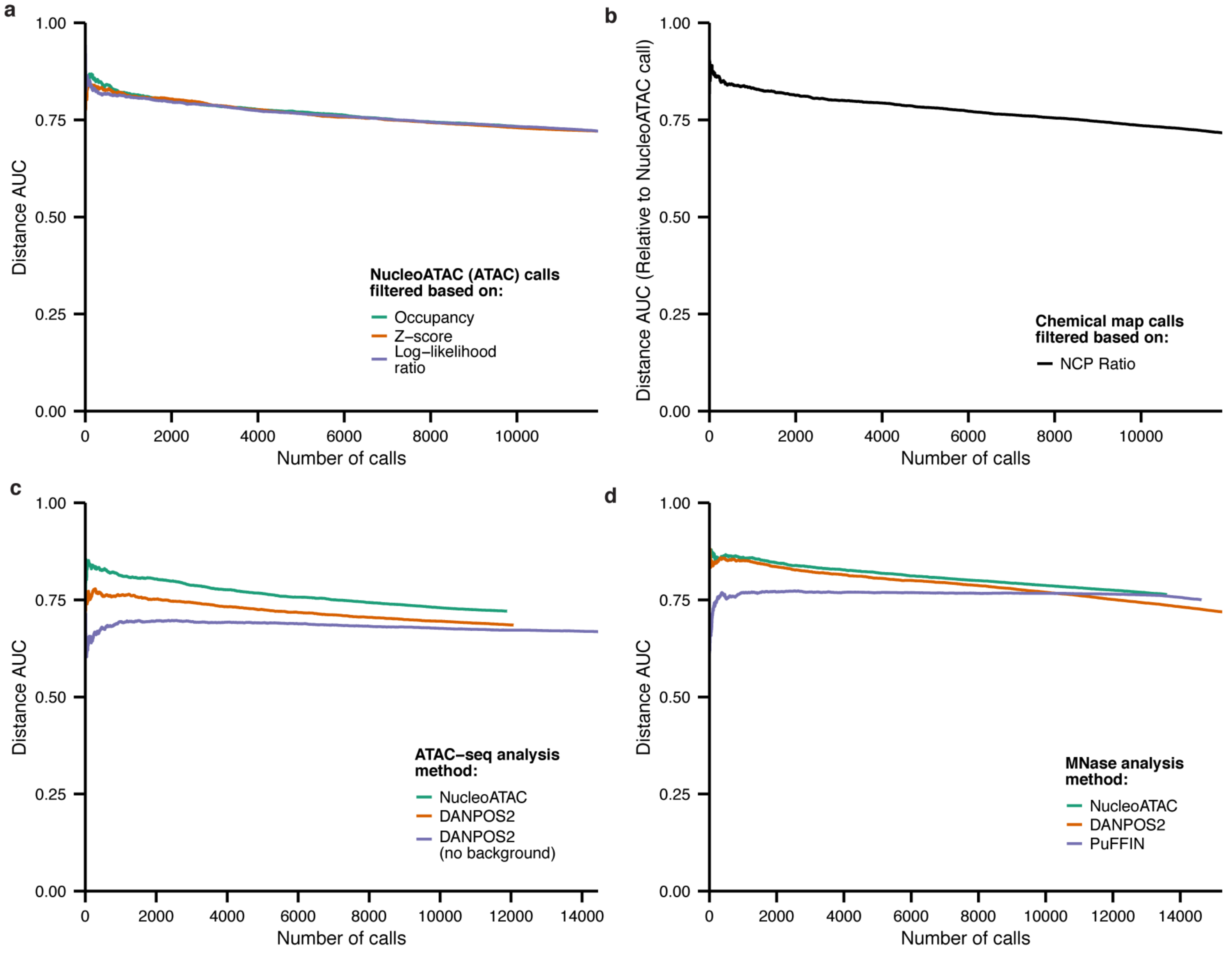

Supplementary Figure 10. Applying more stringent thresholds to either NucleoATAC or chemical mapping calls for *S. cerevisiae* improves concordance between the two sets of calls. A) Distance AUC versus number of NucleoATAC calls made using ATAC-seq when filtering based on NucleoATAC confidence metrics. B) Distance AUC relative to NucleoATAC calls versus number of chemical mapping calls when filtering based on NCP/Noise ratio (as calculated by Brogaard et al. (2012). C) Distance AUC versus number of calls made by different analysis methods for ATAC-seq: NucleoATAC (filtered by Z-score), DANPOS2 (Filtered by Signal), DANPOS2 run without providing short fragments as background (Filtered by Signal) D) Distance AUC versus number of calls made by different analysis methods for MNase: NucleoATAC (filtered by Z-score), DANPOS2 (Filtered by Fuzziness), PuFFIN (filtered by score). For MNase, DANPOS2 calls were filtered by fuzziness while for ATAC-seq they were filtered by signal as fuzziness performed better for MNase but signal performed better for ATAC-seq.

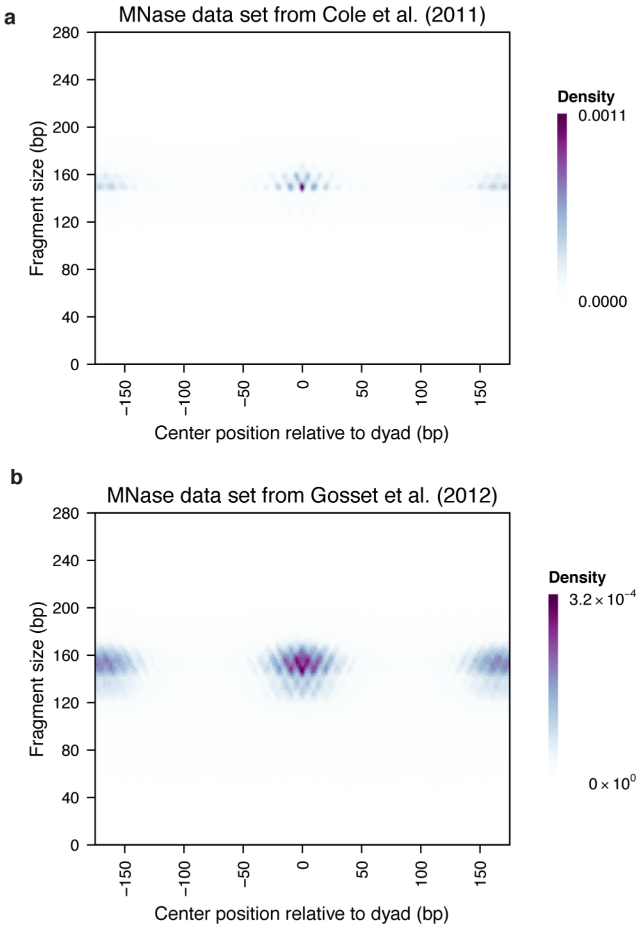

Supplementary Figure 11. MNase V-plots from two different MNase data sets. A) V-Plot based on MNase data from Cole et al. 2011. B) V-Plot based on MNase data set from Gossett and Lieb 2012.

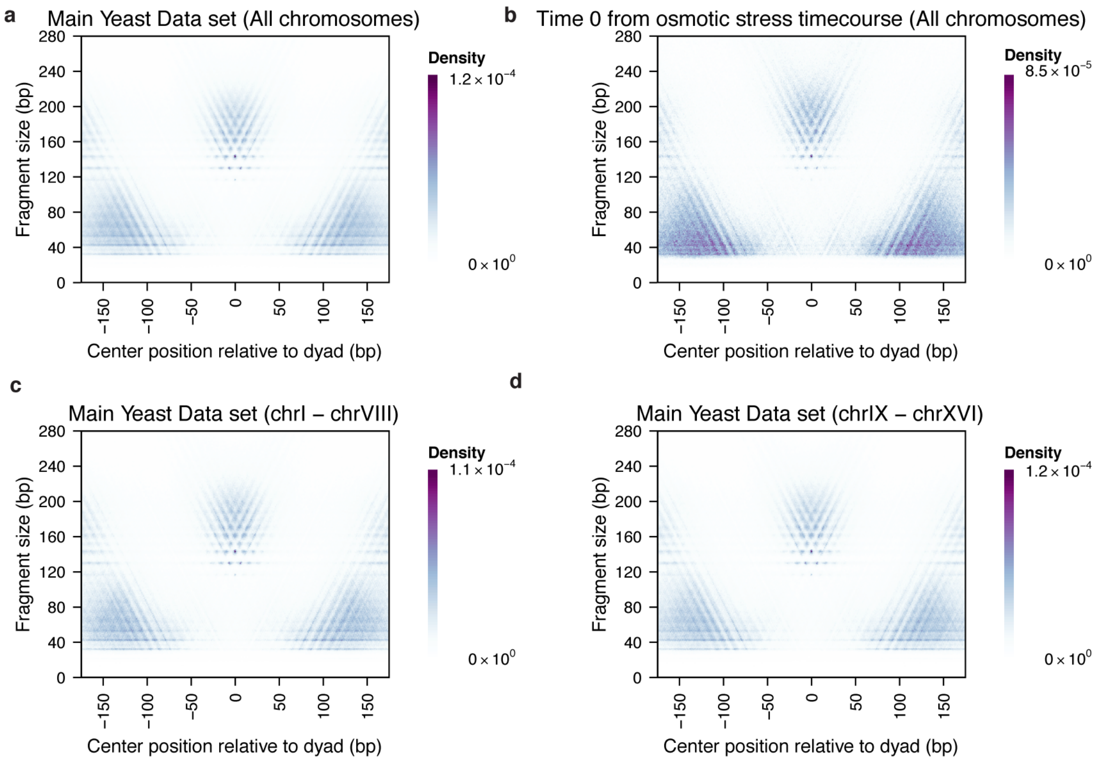

Supplementary Figure 12. V-Plots from different *S. cerevisiae* data samples and different subsets of chemical mapping calls. A) V-Plot from all chemical mapping calls within highly mappable regions of the genome. B) V-plot from same set of chemical mapping calls as A but a different ATAC-seq data set, prepared with different protocol (see **Methods**). C) V-Plot from sub-set of chemical mapping calls used in A, but same ATAC-seq sample. D) V-Plot from different sub-set of chemical mapping calls used in A, but same ATAC-seq sample.

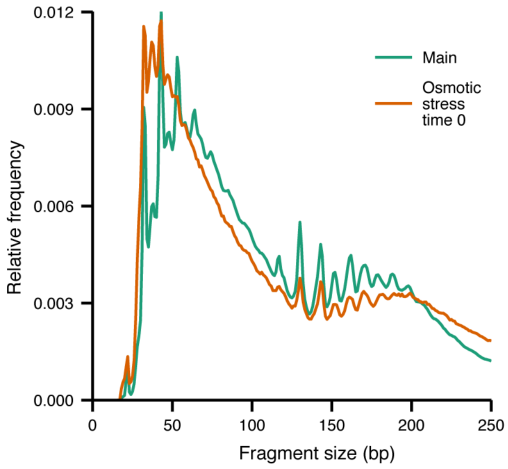

Supplementary Figure 13. Fragment size distributions for two different S. cerevisiae ATAC-seq samples. Samples were prepared from different strains with different spheroplasting and transposition protocols (See Methods).

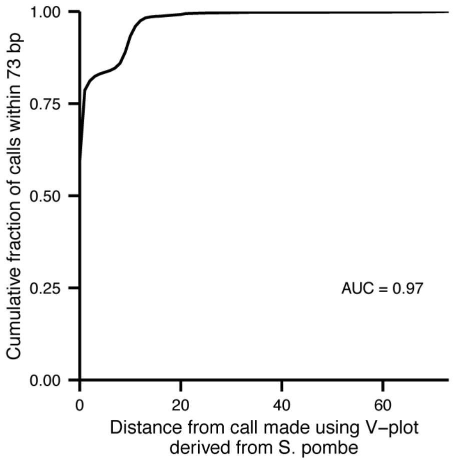

Supplementary Figure 14. Calls using normalized *S. cerevisiae* V-plot are highly similar to calls made using *S. pombe* V-plot. Plot shows cumulative distance of calls made using normalized *S. cerevisiae* V-plot relative to calls made using *S. pombe* V-plot.

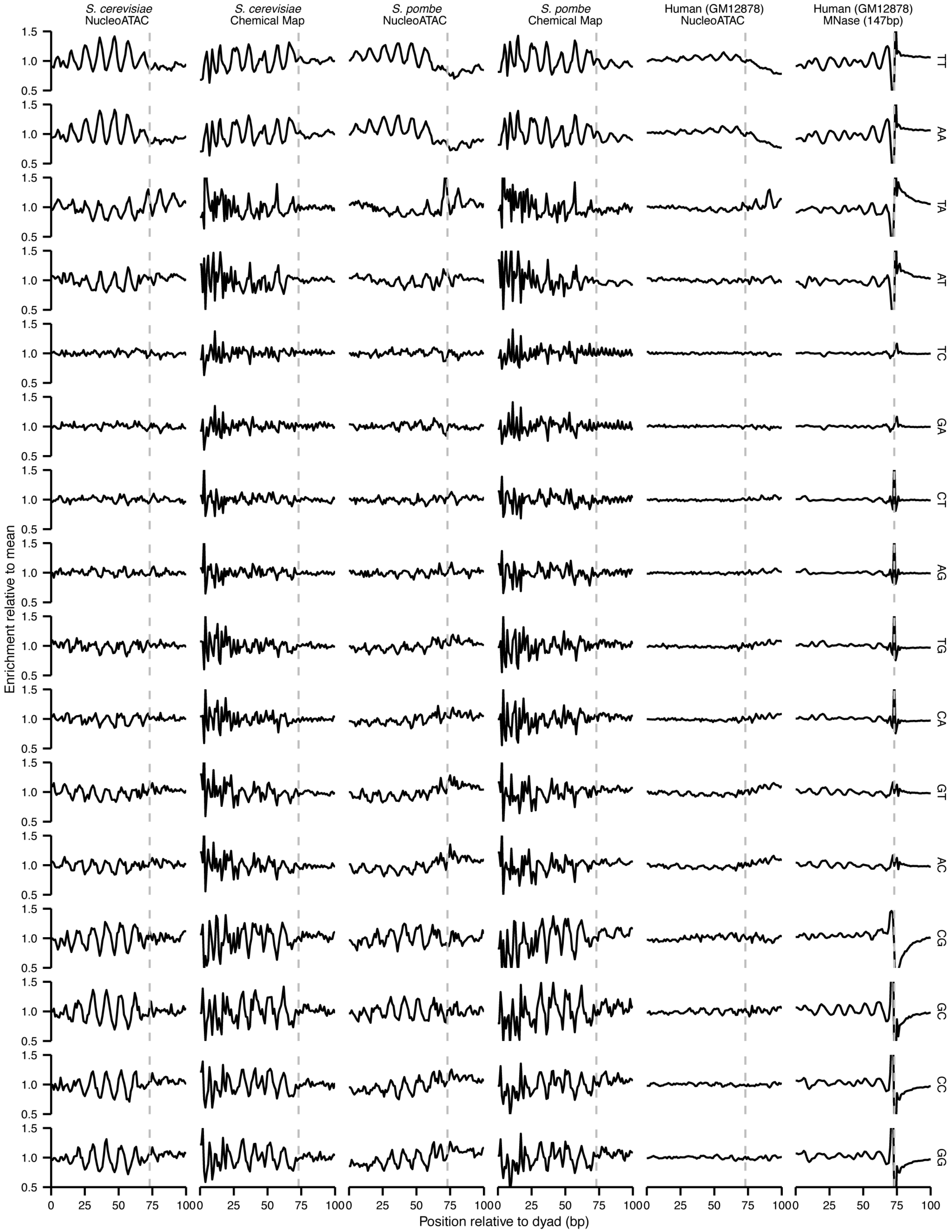

Supplementary Figure 15. Dinucleotide frequency patterns around NucleoATAC calls and chemical mapping calls or 147 bp MNase fragment centers for *S. cerevisiae*, *S. pombe*, and Human (GM12878) within broad open chromatin regions. The dashed gray line at 73 bp indicates the boundary of the DNA around the nucleosome core.

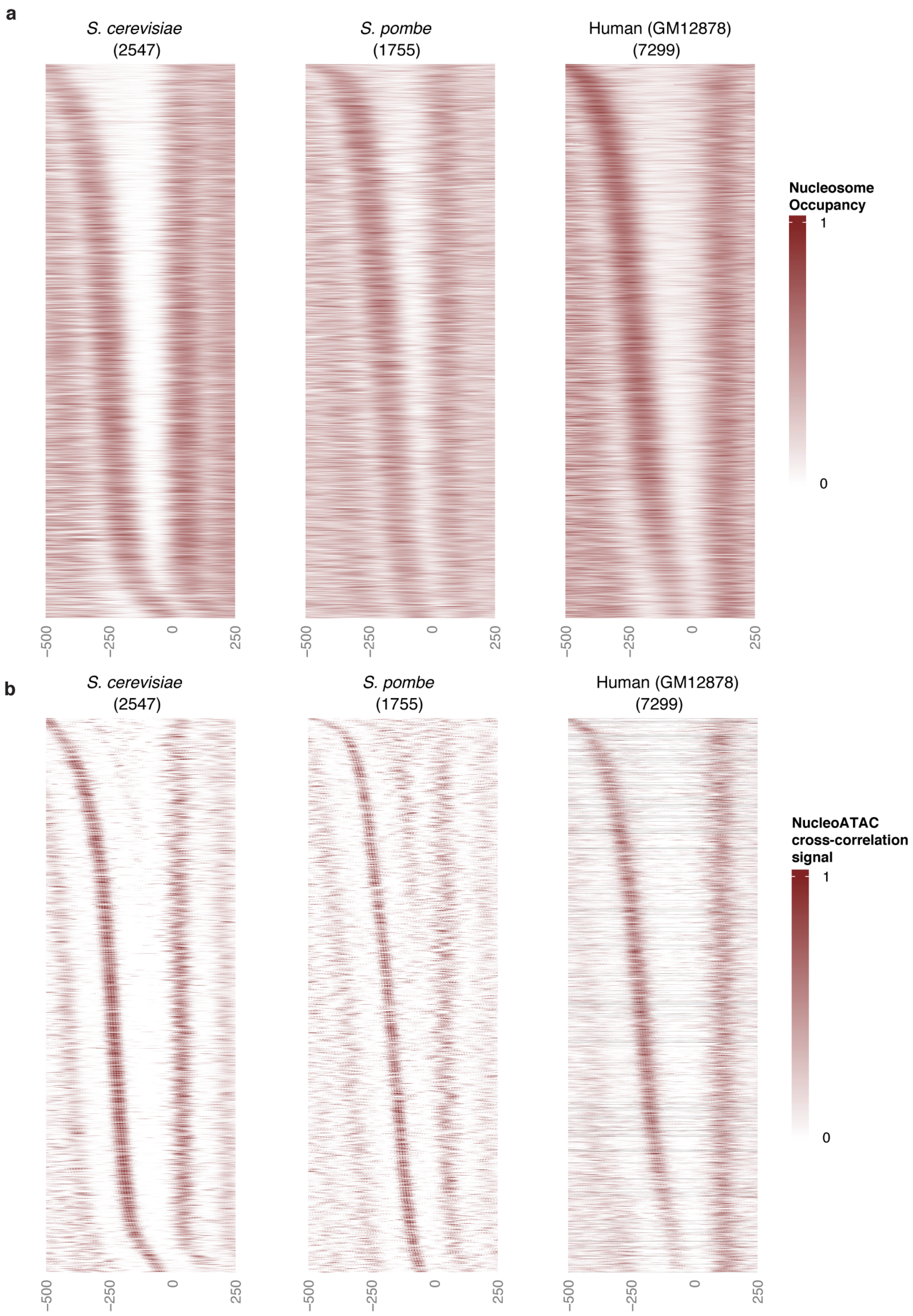

Supplementary Figure 16. High degree of concordance in nucleosome positioning between transcription start sites. A) Nucleosome occupancy at transcription start sites in open chromatin regions for *S. cerevisiae, S. pombe*, and human. B) NucleoATAC normalized cross-correlation signal at transcription start sites in open chromatin regions for *S. cerevisiae, S. pombe*, and human. Signal for each TSS is normalized so max signal is 1.

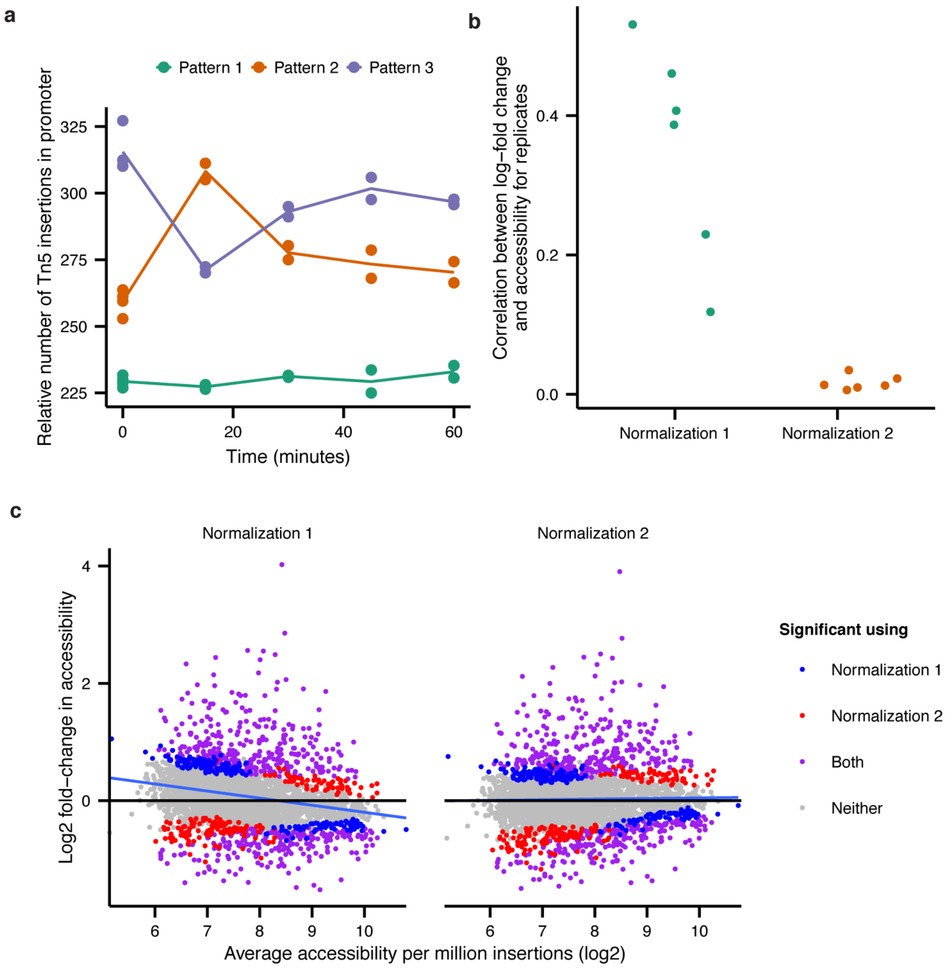

Supplementary Figure 17. Osmotic stress normalization. A) Relative promoter accessibility for genes classified by expression pattern(Ni et al. 2009). Expression pattern 1 corresponds to increased steady expression, pattern 2 to a transient increase in expression, and pattern 3 to a transient decrease in expression. Promoter accessibility is normalized by total number of reads in promoters. B). Correlation between log-fold change in promoter accessibility for replicates and average accessibility when using either normalization based on total reads in promoters (normalization 1) or normalizing based on the distribution of chromatin accessibility of promoters with expression pattern 1 (normalization 2; for more details, see Methods). C) MA plots using Normalization 1 or Normalization 2. Purple points are called significant using either normalization; blue points are only called significant using Normalization1; and red points are called significant using either normalization.

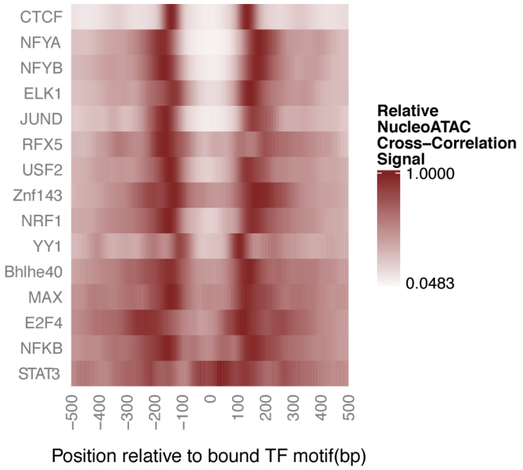

Supplementary Figure 18. NucleoATAC cross-correlation signal around bound TF motifs. Signal value for each row is normalized so maximum is 1.

Supplementary References

Adey A, Morrison HG, Asan, Xun X, Kitzman JO, Turner EH, Stackhouse B, MacKenzie AP, Caruccio NC, Zhang X, et al. 2010. Rapid, low-input, low-bias construction of shotgun fragment libraries by high-density in vitro transposition. *Genome Biol* **11**: R119.

Buenrostro JD, Wu B, Litzenburger UM, Ruff D, Gonzales ML, Snyder MP, Chang HY, Greenleaf WJ. 2015. Single-cell chromatin accessibility reveals principles of regulatory variation. *Nature*.

Cole HA, Howard BH, Clark DJ. 2011. Activation-induced disruption of nucleosome position clusters on the coding regions of Gcn4-dependent genes extends into neighbouring genes. *Nucleic Acids Res* **39**: 9521–35.

Gossett AJ, Lieb JD. 2012. In vivo effects of histone H3 depletion on nucleosome occupancy and position in Saccharomyces cerevisiae. *PLoS Genet* **8**: e1002771.

Ni L, Bruce C, Hart C, Leigh-Bell J, Gelperin D, Umansky L, Gerstein MB, Snyder M. 2009. Dynamic and complex transcription factor binding during an inducible response in yeast. *Genes Dev* **23**: 1351–63.
